# Supplementary figures and images for: OpenABM-Covid19—An agent-based model for non-pharmaceutical interventions against COVID-19 including contact tracing
Source: PLoS Comput Biol. 2021 Jul 12;17(7):e1009146. doi: 10.1371/journal.pcbi.1009146 (PMC8328312; doi:10.1371/journal.pcbi.1009146)

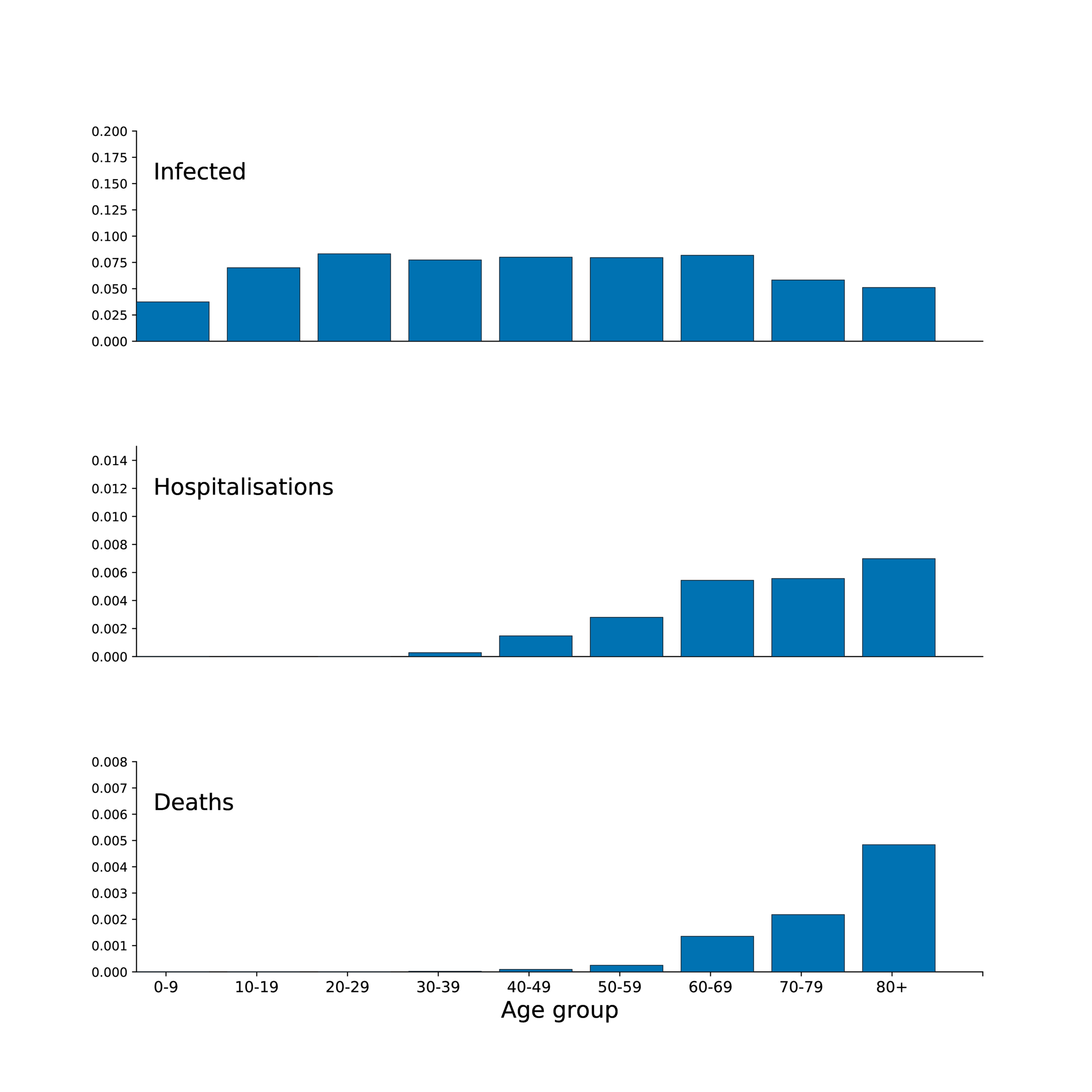

Supplement: S1 Fig — Simulations are from the end of a single simulation in a population of 1 million individuals with UK-like demographics and control interventions. The simulation was run for 77 days after lockdown started. The denominator in each calculation is the number of individuals in each age group in the total population (e.g. of the total population, the middle panel shows the proportion of each age group that was hospitalised in the simulation). (TIF) [file pcbi.1009146.s001.tif]

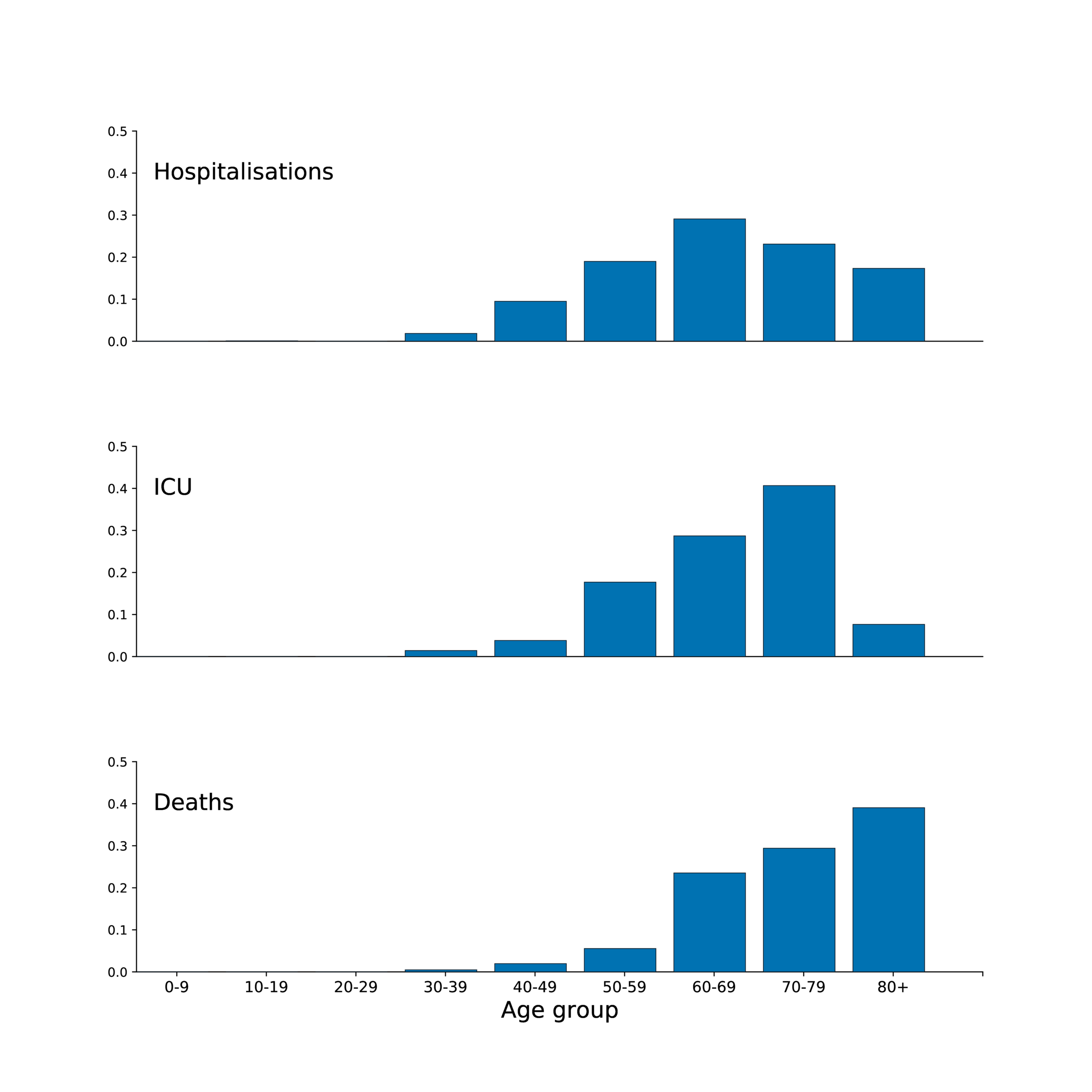

Supplement: S2 Fig — Data are from a single simulation of 1 million individuals with UK-like demographics and control interventions. The simulation was run for 77 days after lockdown started. The denominator is the number of individuals ever having been in the state in question (e.g. of all simulated hospitalisations, the top panel shows the distribution of these by age). (TIF) [file pcbi.1009146.s002.tif]

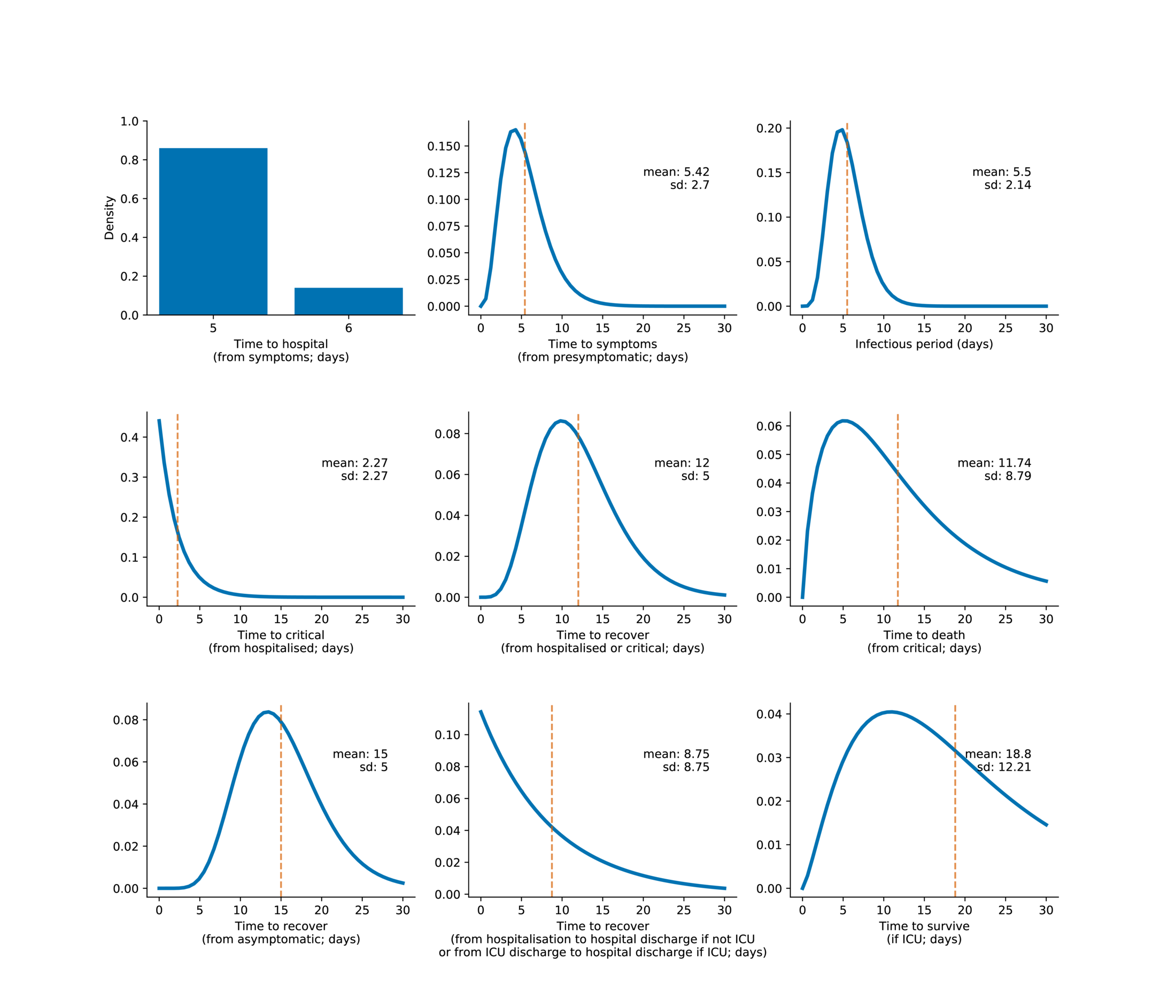

Supplement: S3 Fig — All distributions are gamma except time to hospital which is a shifted Bernoulli distribution. Mean of each gamma distribution is shown with a vertical dashed line. (TIF) [file pcbi.1009146.s003.tif]

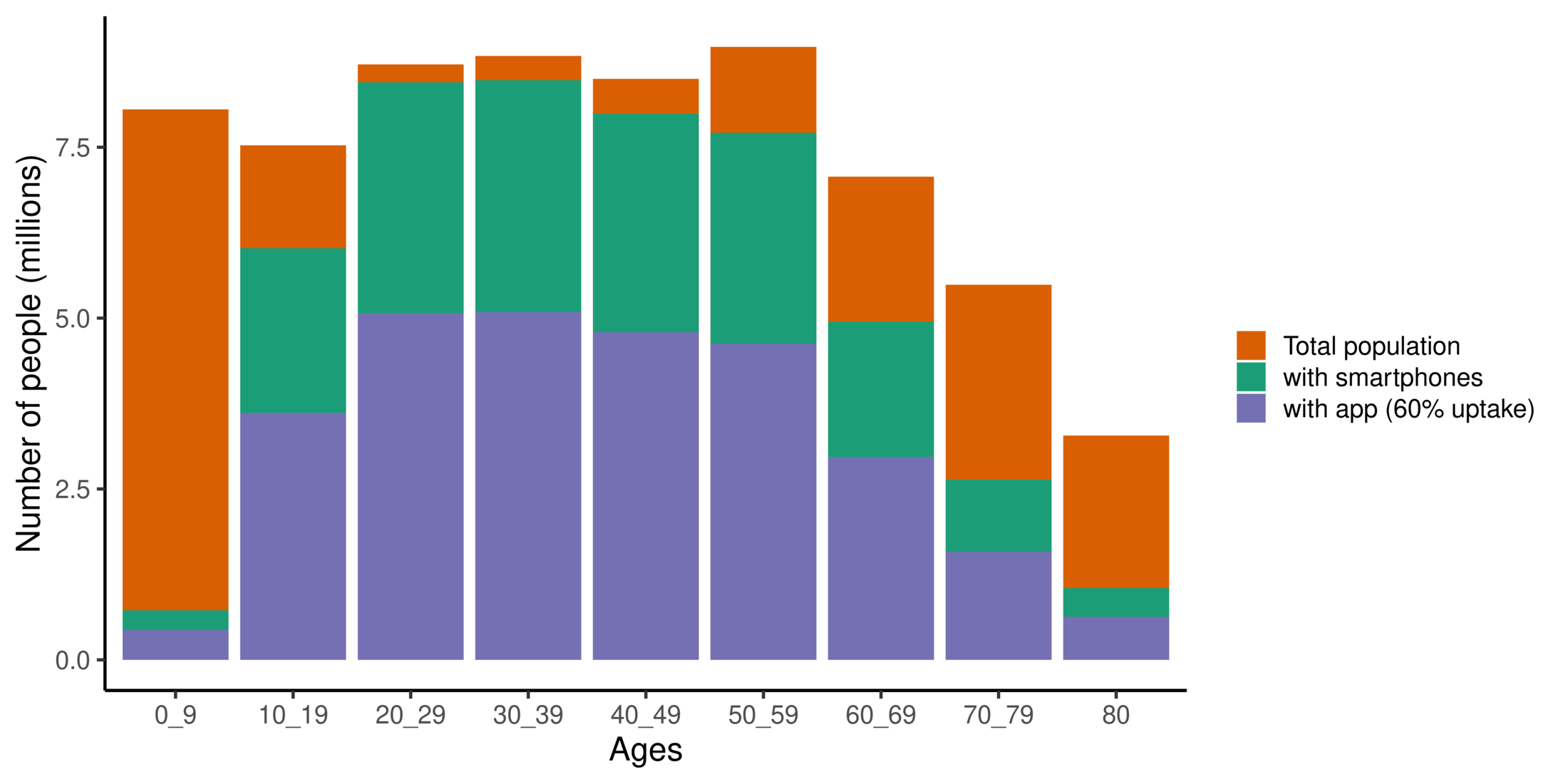

Supplement: S4 Fig — (TIF) [file pcbi.1009146.s004.tif]

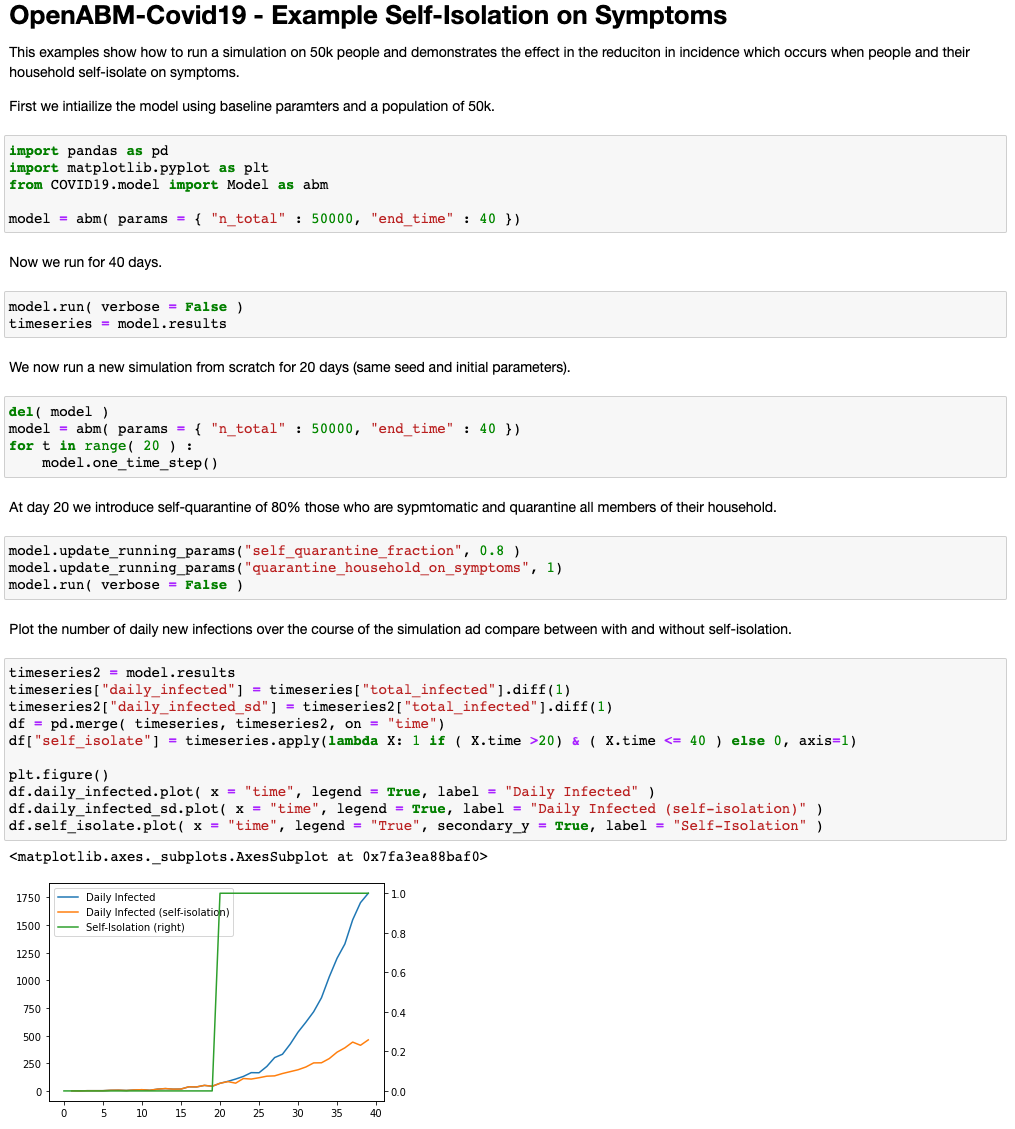

Supplement: S5 Fig — Code for this notebook is provided at https://github.com/BDI-pathogens/OpenABM-Covid19-model-paper/blob/master/notebooks/example_self_isolation.ipynb. (TIFF) [file pcbi.1009146.s005.tiff]

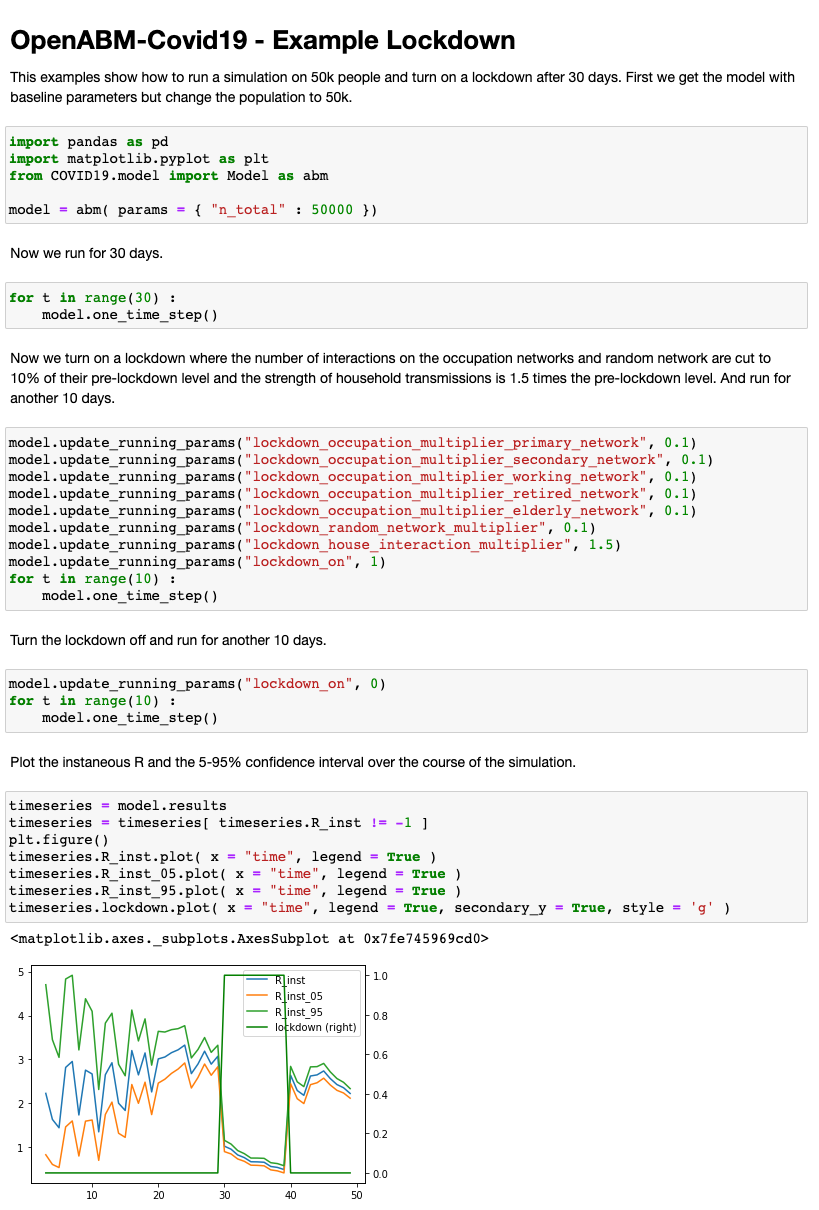

Supplement: S6 Fig — The Python code used for this simulation, the code is also at https://github.com/BDI-pathogens/OpenABM-Covid19-model-paper/blob/master/notebooks/example_lockdown.ipynb. (TIFF) [file pcbi.1009146.s006.tiff]

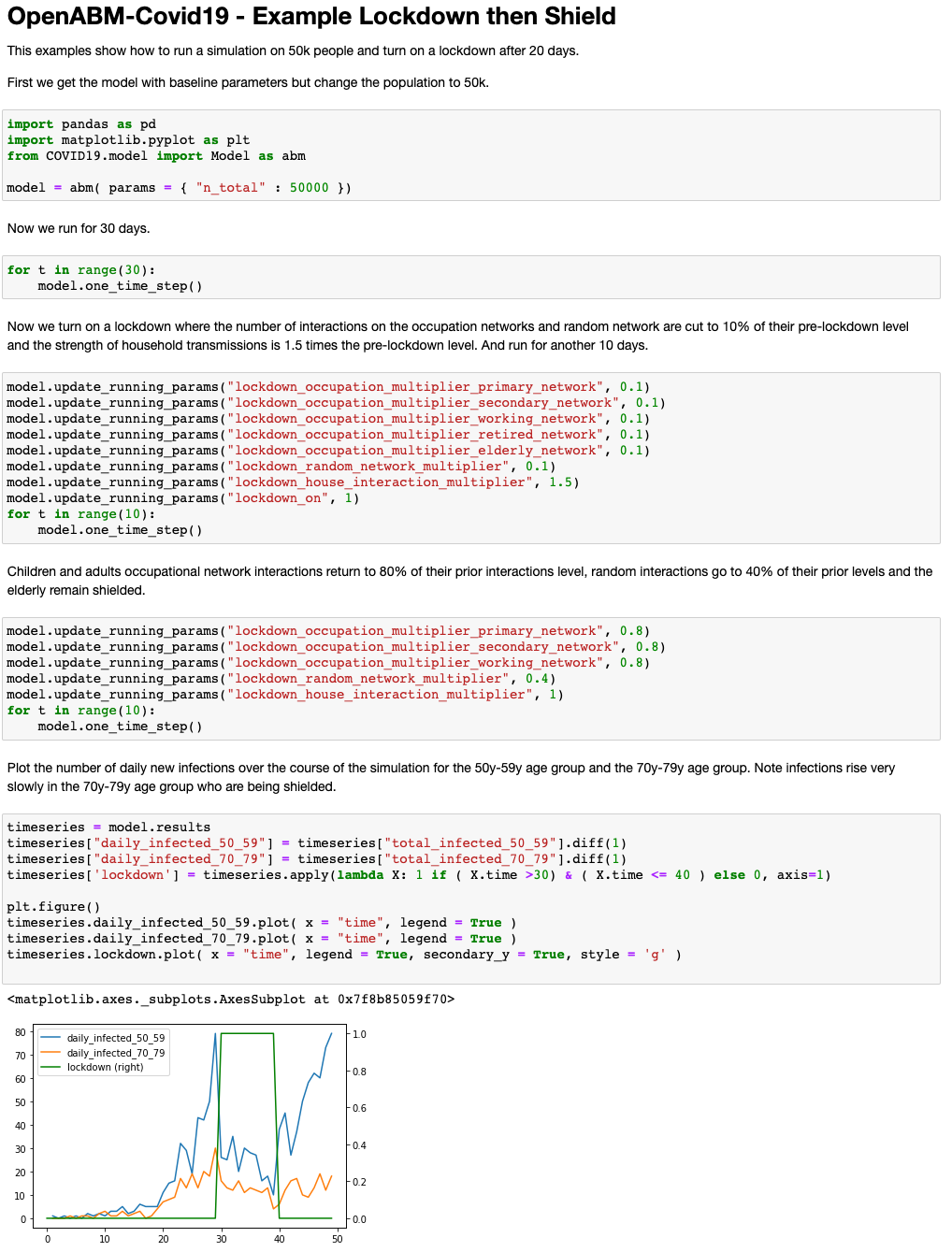

Supplement: S7 Fig — Code for this notebook is provided at https://github.com/BDI-pathogens/OpenABM-Covid19-model-paper/blob/master/notebooks/example_lockdown_shield.ipynb. (TIFF) [file pcbi.1009146.s007.tiff]

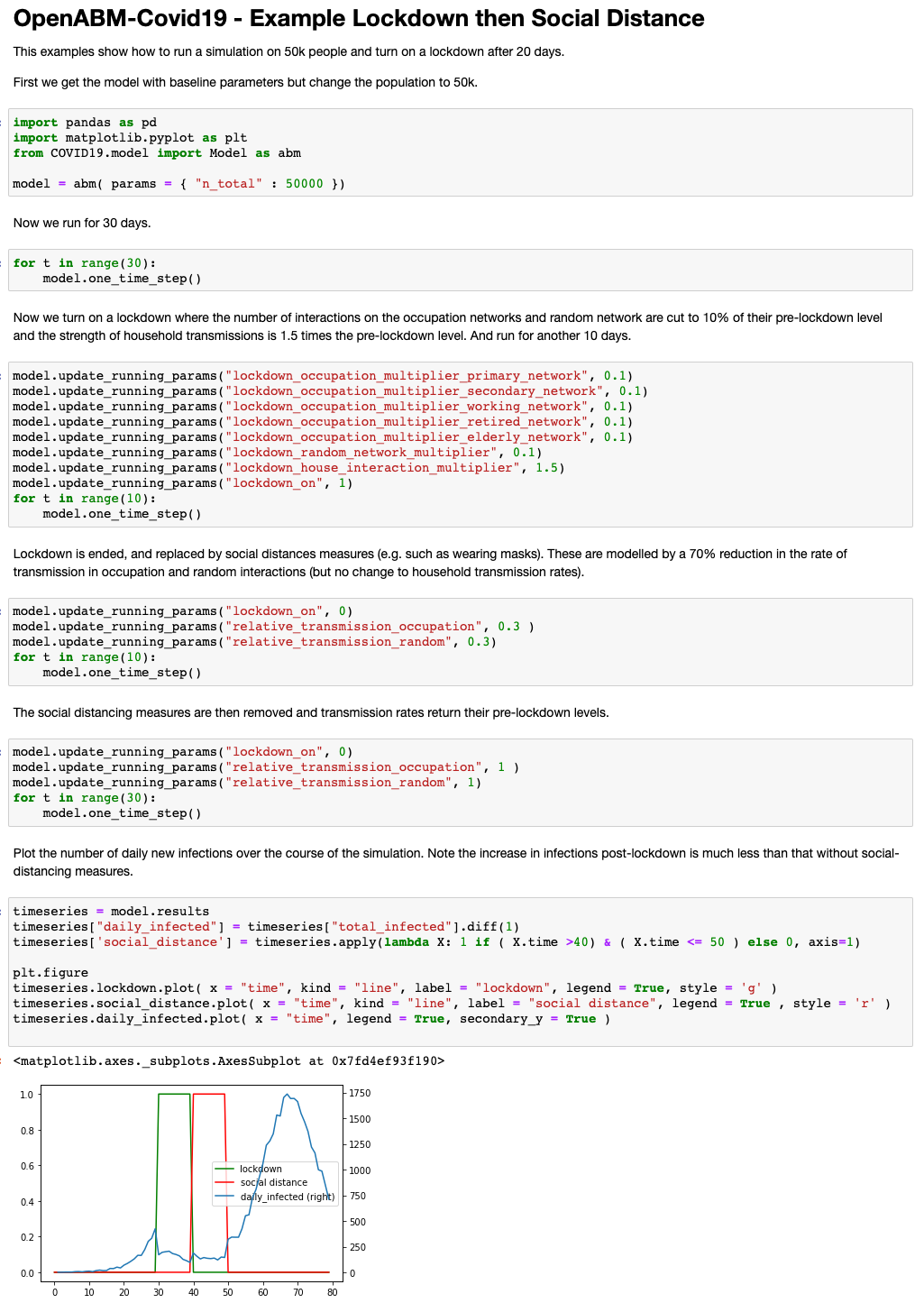

Supplement: S8 Fig — Code for this notebook is provided at https://github.com/BDI-pathogens/OpenABM-Covid19-model-paper/blob/master/notebooks/example_lockdown_social_distance.ipynb. (TIFF) [file pcbi.1009146.s008.tiff]

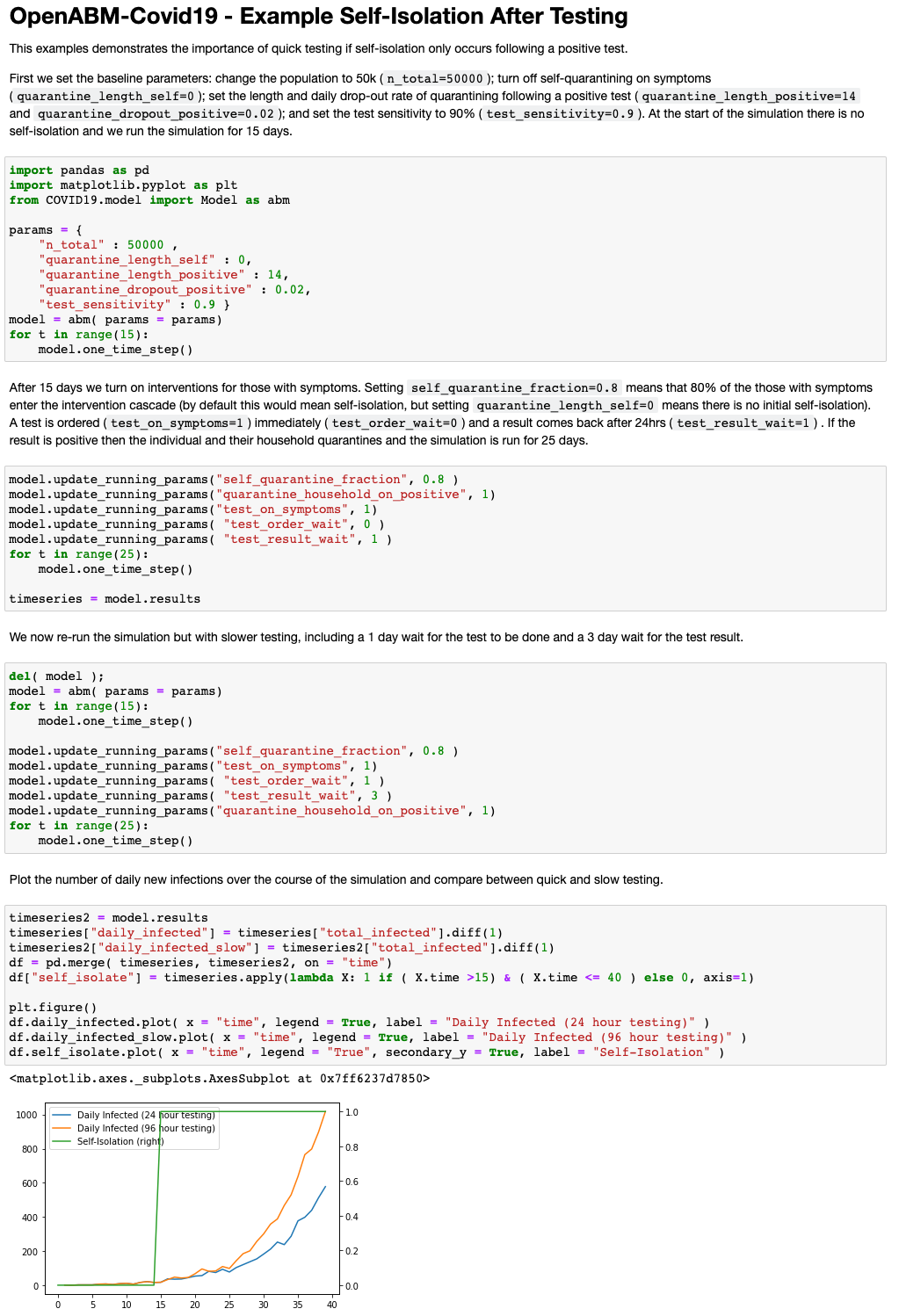

Supplement: S9 Fig — Code for this notebook is provided at https://github.com/BDI-pathogens/OpenABM-Covid19-model-paper/blob/master/notebooks/example_testing.ipynb. (TIFF) [file pcbi.1009146.s009.tiff]

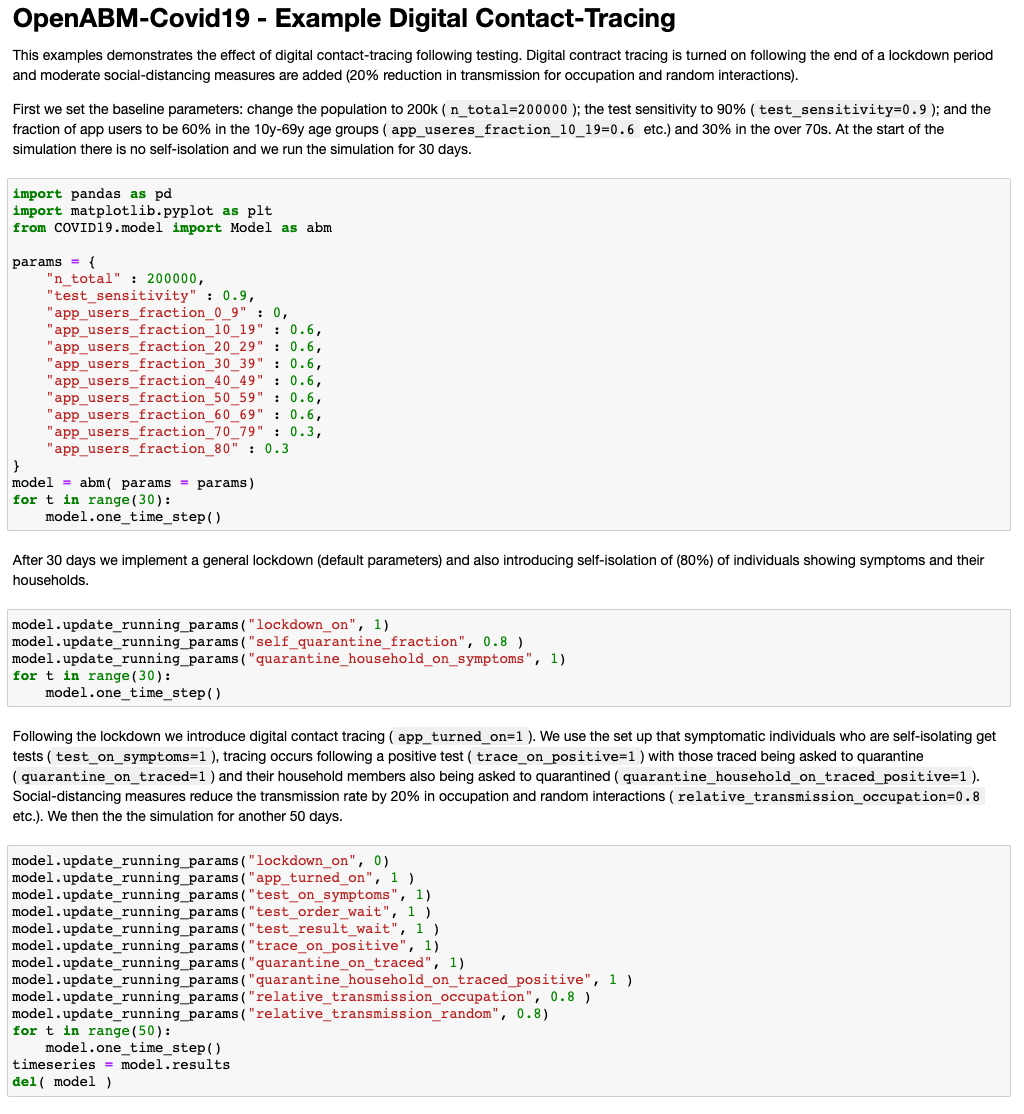

Supplement: S10 Fig — Code for this notebooks is provided at https://github.com/BDI-pathogens/OpenABM-Covid19-model-paper/blob/master/notebooks/example_digital_contact_tracing.ipynb. (TIFF) [file pcbi.1009146.s010.tiff]

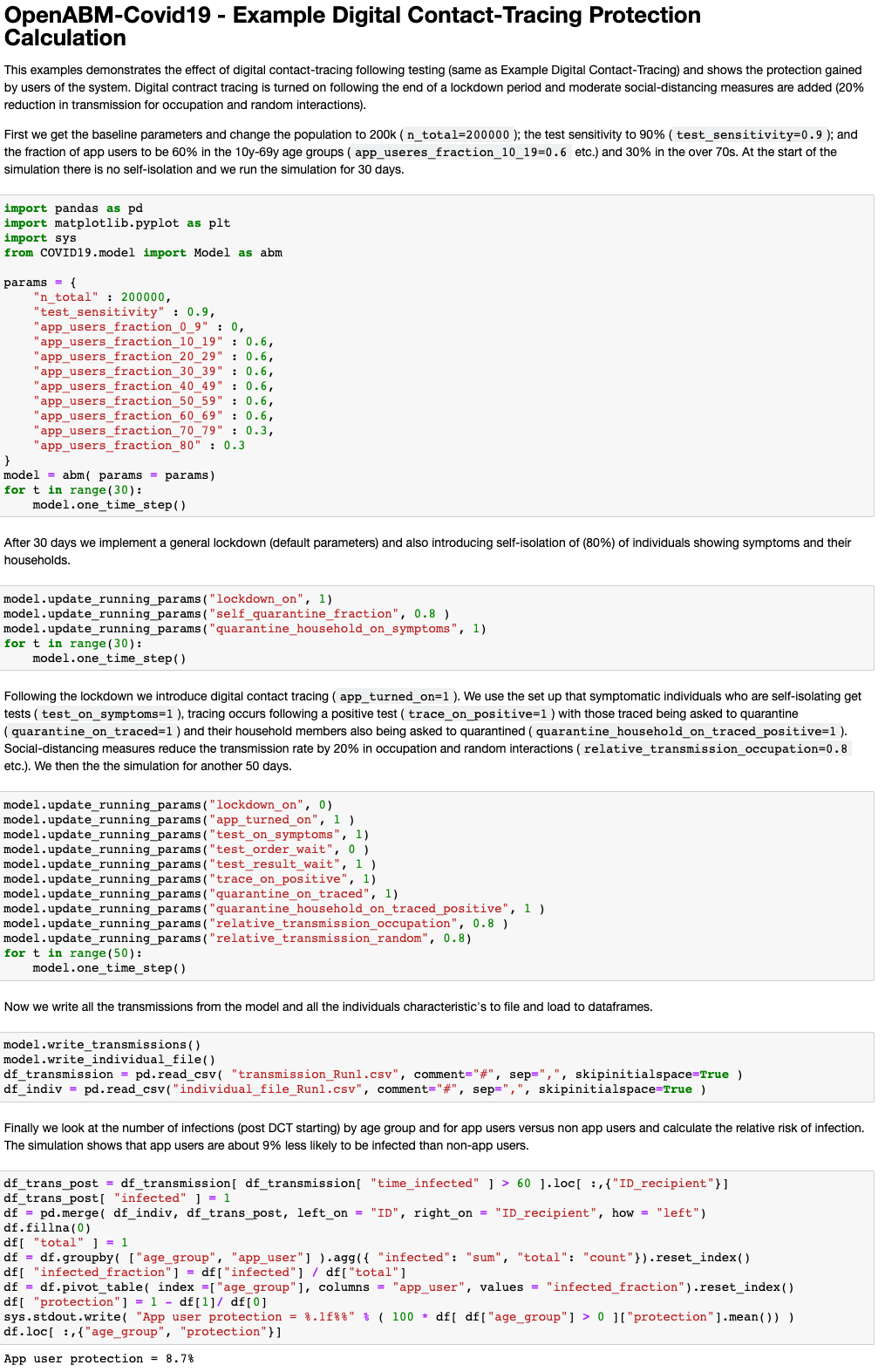

Supplement: S11 Fig — Code for this notebook is provided at https://github.com/BDI-pathogens/OpenABM-Covid19-model-paper/blob/master/notebooks/example_digital_contact_tracing_protect.ipynb. (TIFF) [file pcbi.1009146.s011.tiff]

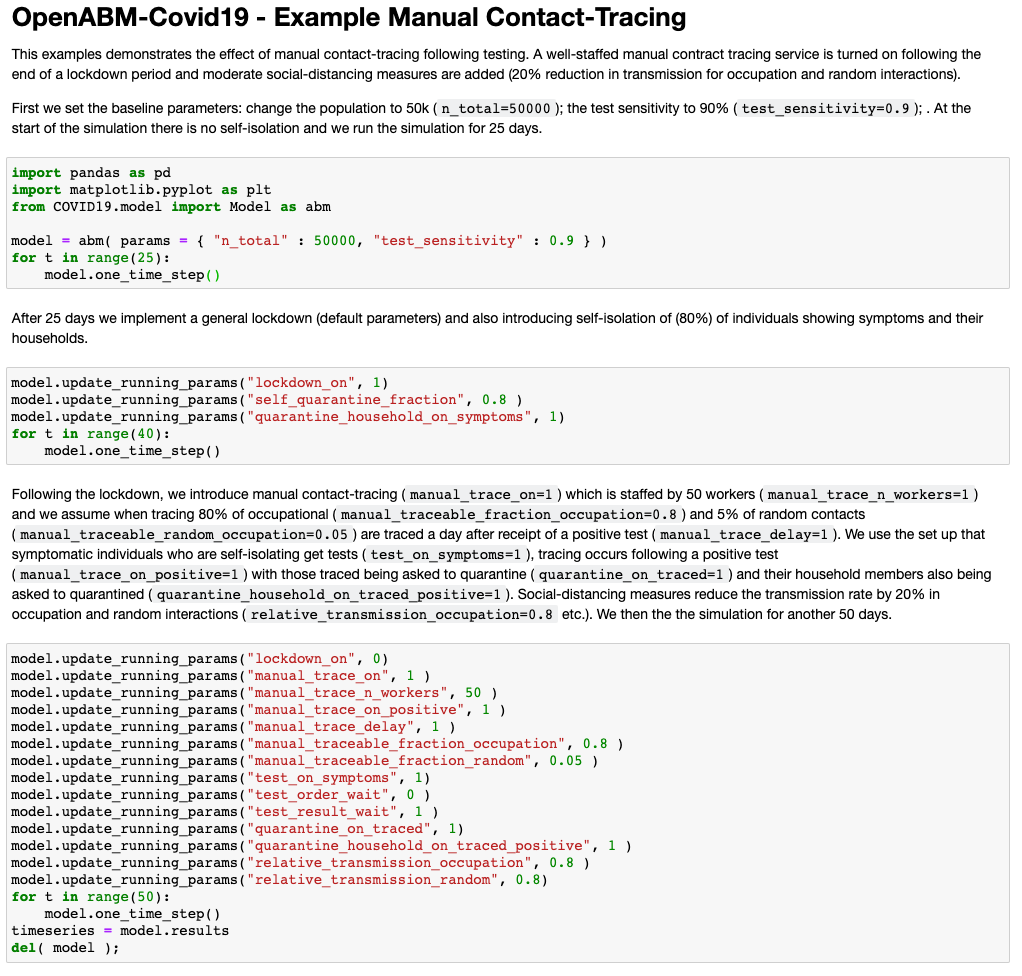

Supplement: S12 Fig — Code for this notebook is provided at https://github.com/BDI-pathogens/OpenABM-Covid19-model-paper/blob/master/notebooks/example_manual_contact_tracing.ipynb. (TIFF) [file pcbi.1009146.s012.tiff]

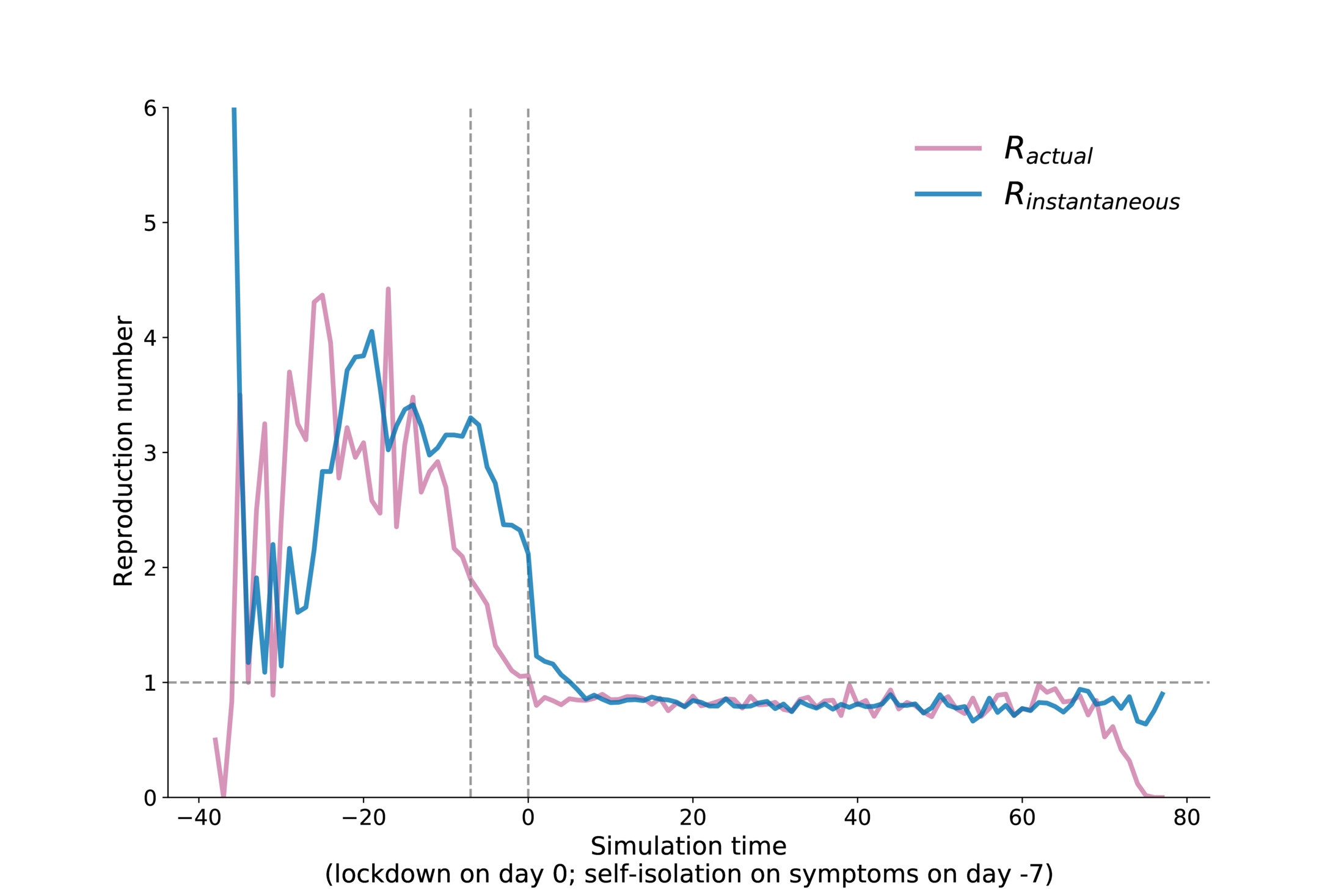

Supplement: S13 Fig — Data from a single simulated outbreak with R calculated using the complete simulated transmission tree (actual) or using the time series (instantaneous). Simulation data are for a single simulation in a population of 1 million individuals with UK-like demographics. The vertical dashed mark where interventions were introduced (self-isolation on symptoms followed by lockdown), note that Ractual is reduced prior to the introduction of each intervention. (TIF) [file pcbi.1009146.s013.tif]

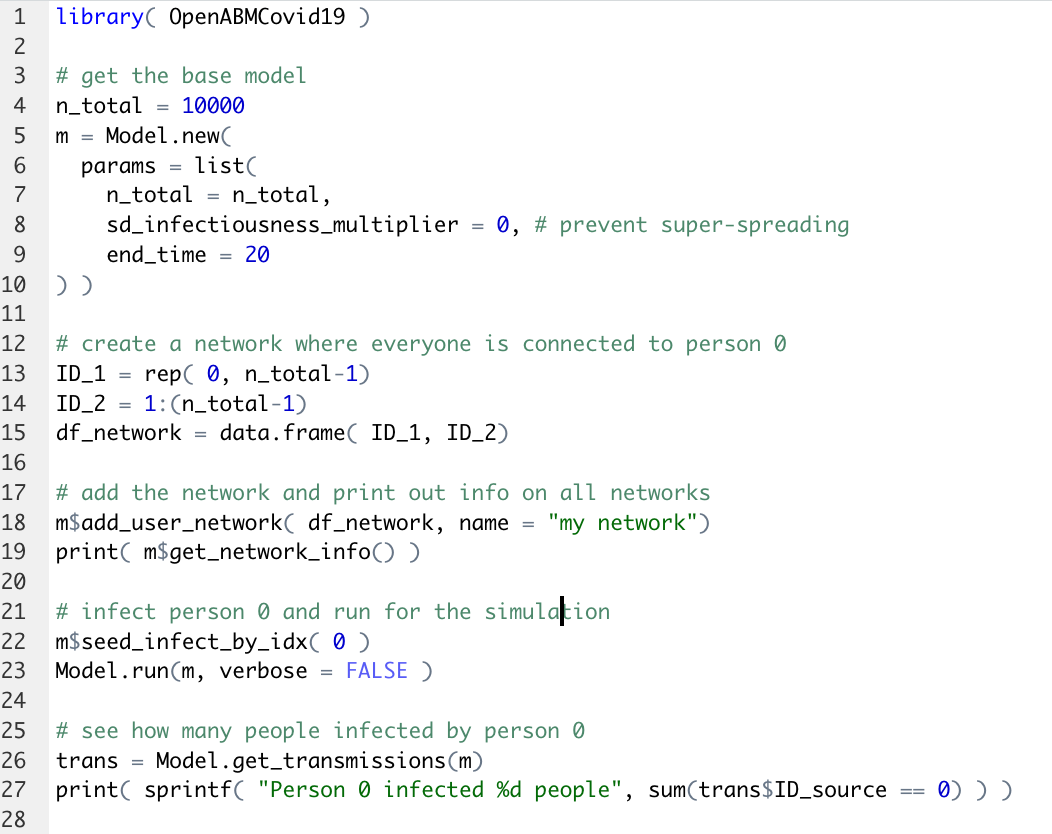

Supplement: S14 Fig — R script demonstrating how to add a user specified network. The R code is at: https://github.com/BDI-pathogens/OpenABM-Covid19-model-paper/blob/master/R/figS14_example_add_network.R. (TIFF) [file pcbi.1009146.s014.tiff]

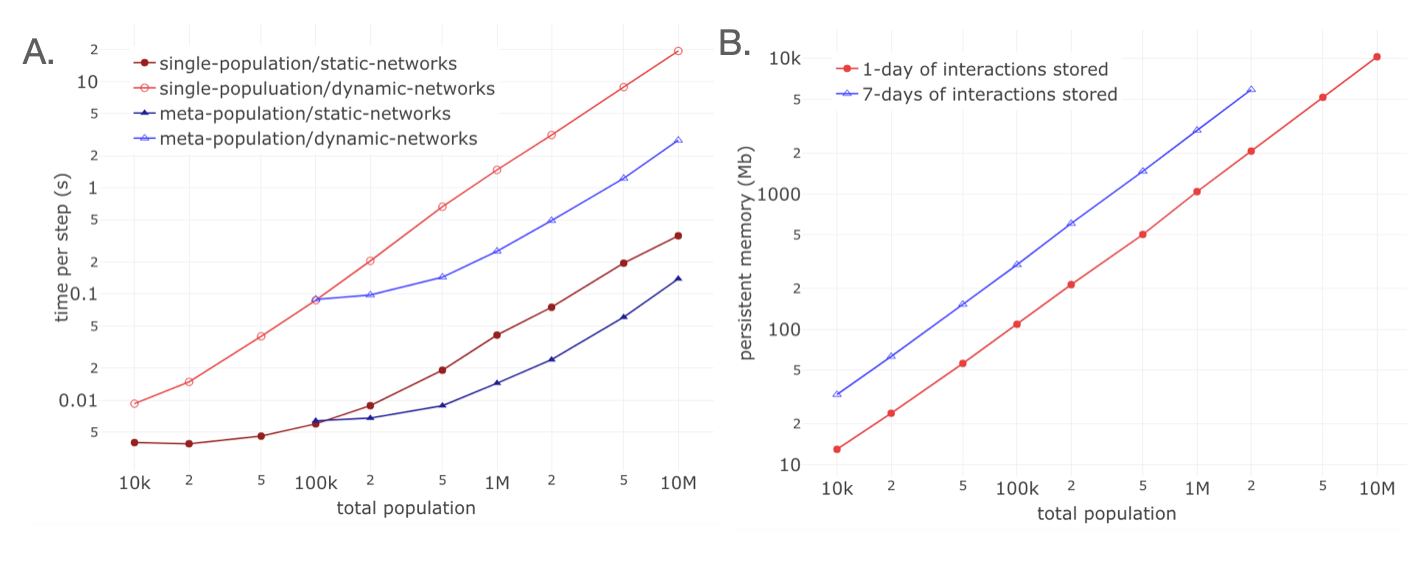

Supplement: S15 Fig — A. The computation time per day for different size populations. The default networks are dynamic and are rebuilt each day, whereas the static networks are not changed after the first day. The meta-population model was run on a quad-core processor. B. The required memory for a simulation which is linear in population. (TIFF) [file pcbi.1009146.s015.tiff]

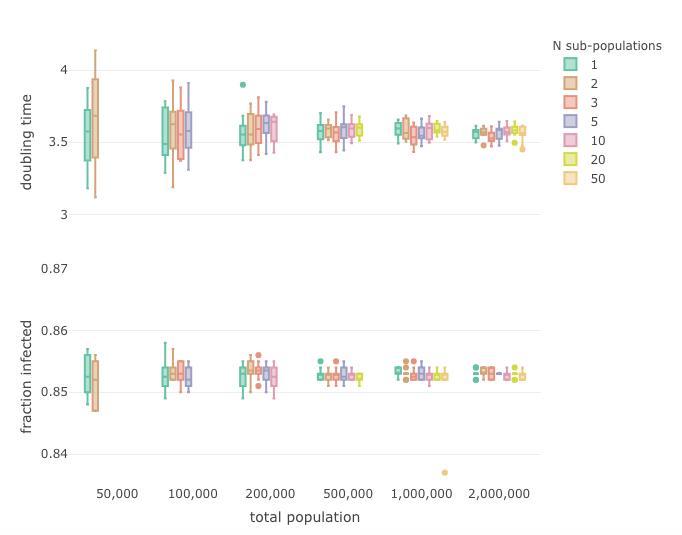

Supplement: S16 Fig — Simulations for epidemics were run for different population sizes and split into different numbers of equal sub-populations in meta-models (zero case migration). Initially the epidemic was seeded with 0.05% infections and an uncontrolled epidemic was allowed to develop for 100 days, with approximately no new infections at the end. A minimum of 20k people was required in each subpopulation in order for there to be sufficient seed infections to prevent a stochastic extinction at the start. Each simulated epidemic was characterised by 2 basic statistics: the doubling time in days to go from 1% to 2% of the population infected; and the total fraction of the population infected. Each configuration was run 10 times and the figure is a box plot of the results, with the number of subpopulations shown as separate colours. The simulations show that the mean doubling time and fraction infected are roughly independent of the total population size. The stochastic variation is determined by the total population and is independent of the number of subpopulations. With a total population of at least 1 million people, the stochastic variation in the doubling time was <0.2 days and in the total number infected was <0.5%. (TIFF) [file pcbi.1009146.s016.tiff]

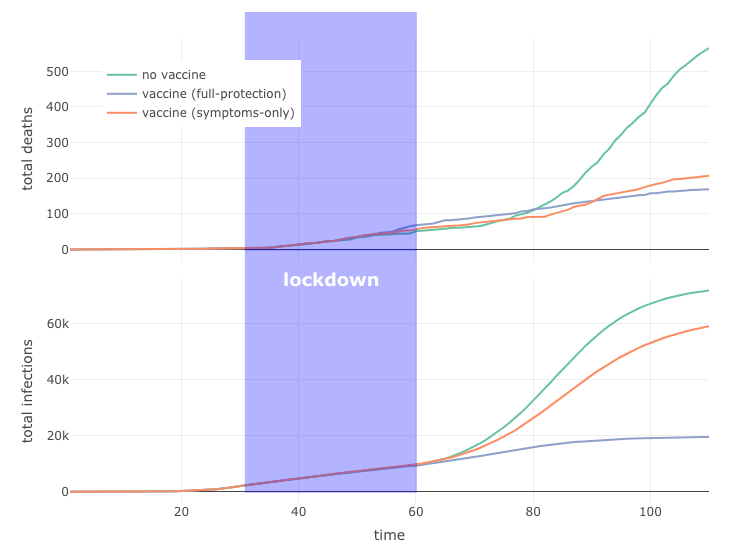

Supplement: S17 Fig — A simulation of a vaccine programme implemented after a lockdown period to control the epidemic. The epidemic was allowed to grow until 2% of the population had been infected, at which point a lockdown was implemented for 30 days along with a vaccination programme where 2% of adults were inoculated each day (vaccine 90% effective after 15-days). The figure compares the total deaths and infections for a vaccine which offers full protection from symptoms, to one which only offers protection from symptoms and to no vaccine programme. The R code for generating this figure is at https://github.com/BDI-pathogens/OpenABM-Covid19-model-paper/blob/master/R/figS17_vaccine.R. (TIFF) [file pcbi.1009146.s017.tiff]

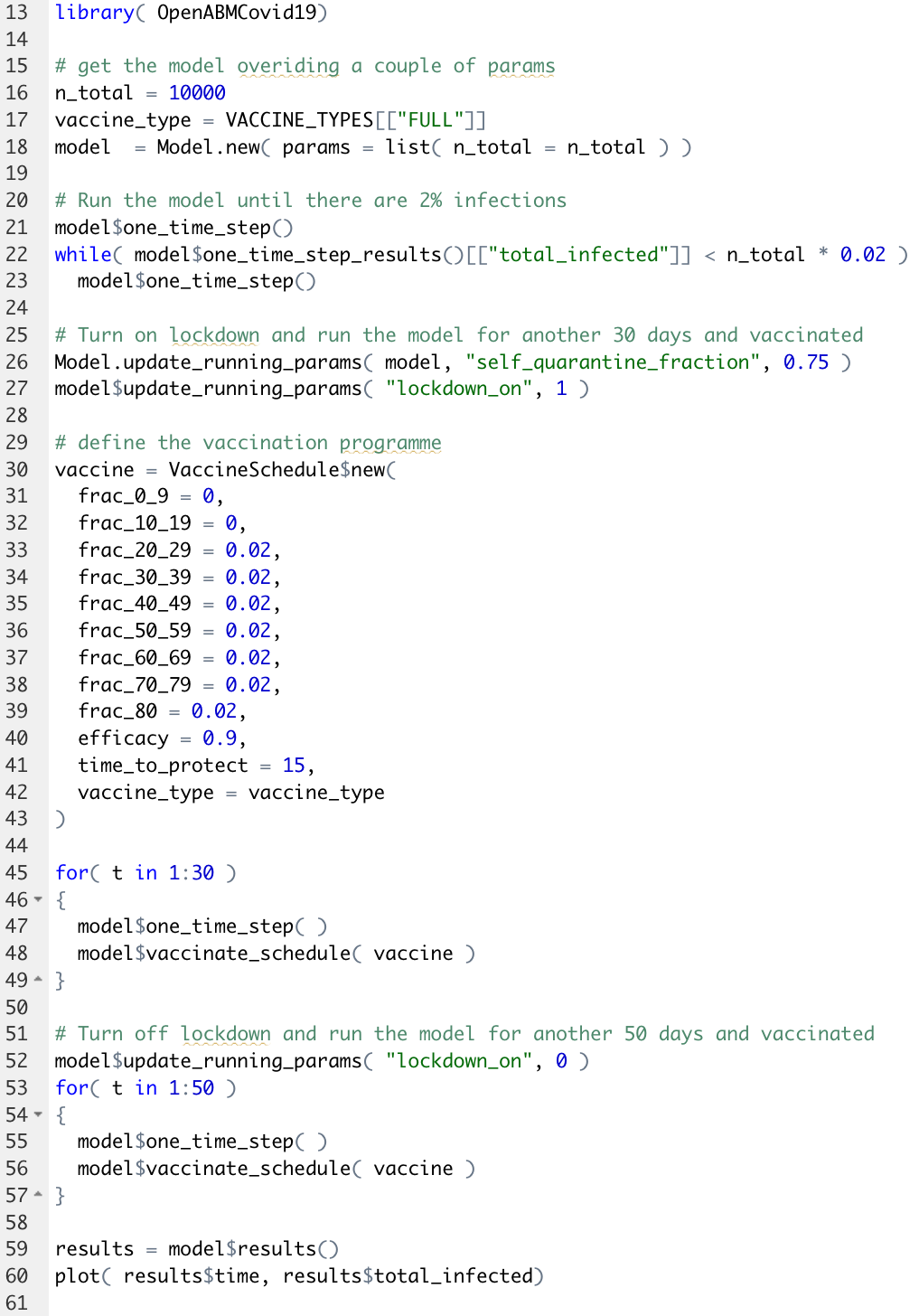

Supplement: S18 Fig — R used for generating the simulations with vaccination programmes. The R code is at https://github.com/BDI-pathogens/OpenABM-Covid19-model-paper/blob/master/R/figS18_example_vaccination.R. (TIFF) [file pcbi.1009146.s018.tiff]

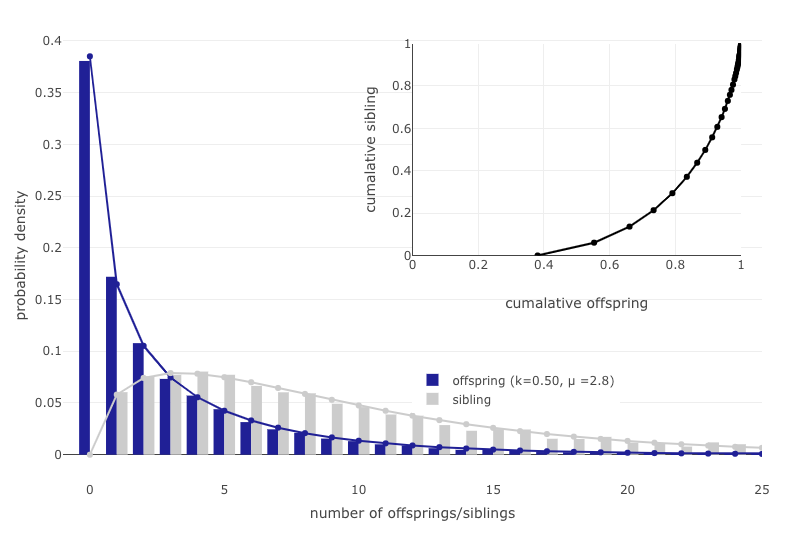

Supplement: S19 Fig — The offspring distribution (in blue) and the sibling distribution (in grey). A negative-binomial fitted to the offspring distribution gives the estimate of k = 0.51. The inset is the cumulative sibling distribution against the cumulative offspring distribution and shows that the 70% of infections are generated by the top 20% of individuals. (TIFF) [file pcbi.1009146.s019.tiff]

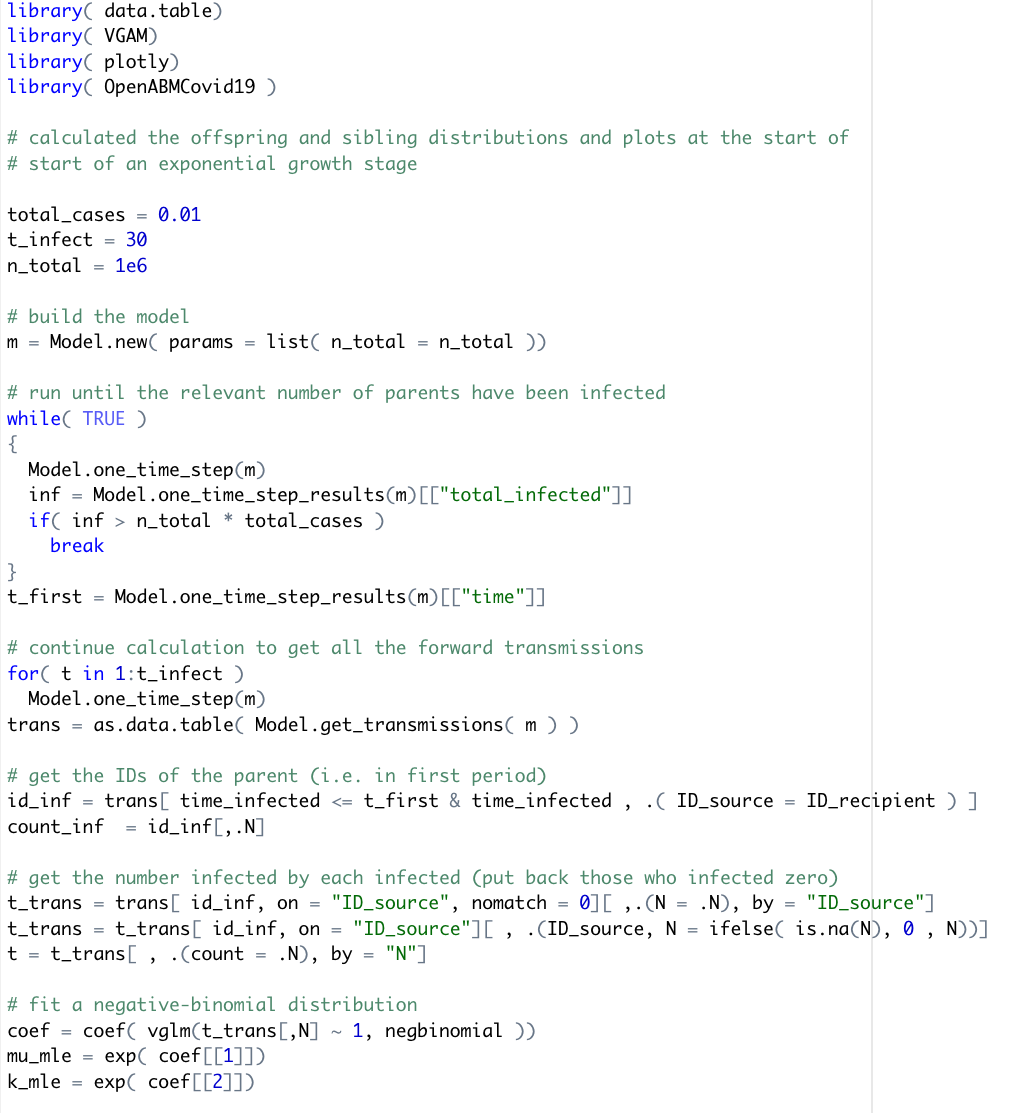

Supplement: S20 Fig — R script used for generating the offspring distribution is at https://github.com/BDI-pathogens/OpenABM-Covid19-model-paper/blob/master/R/figS20-offspring-distribution.R. (TIFF) [file pcbi.1009146.s020.tiff]

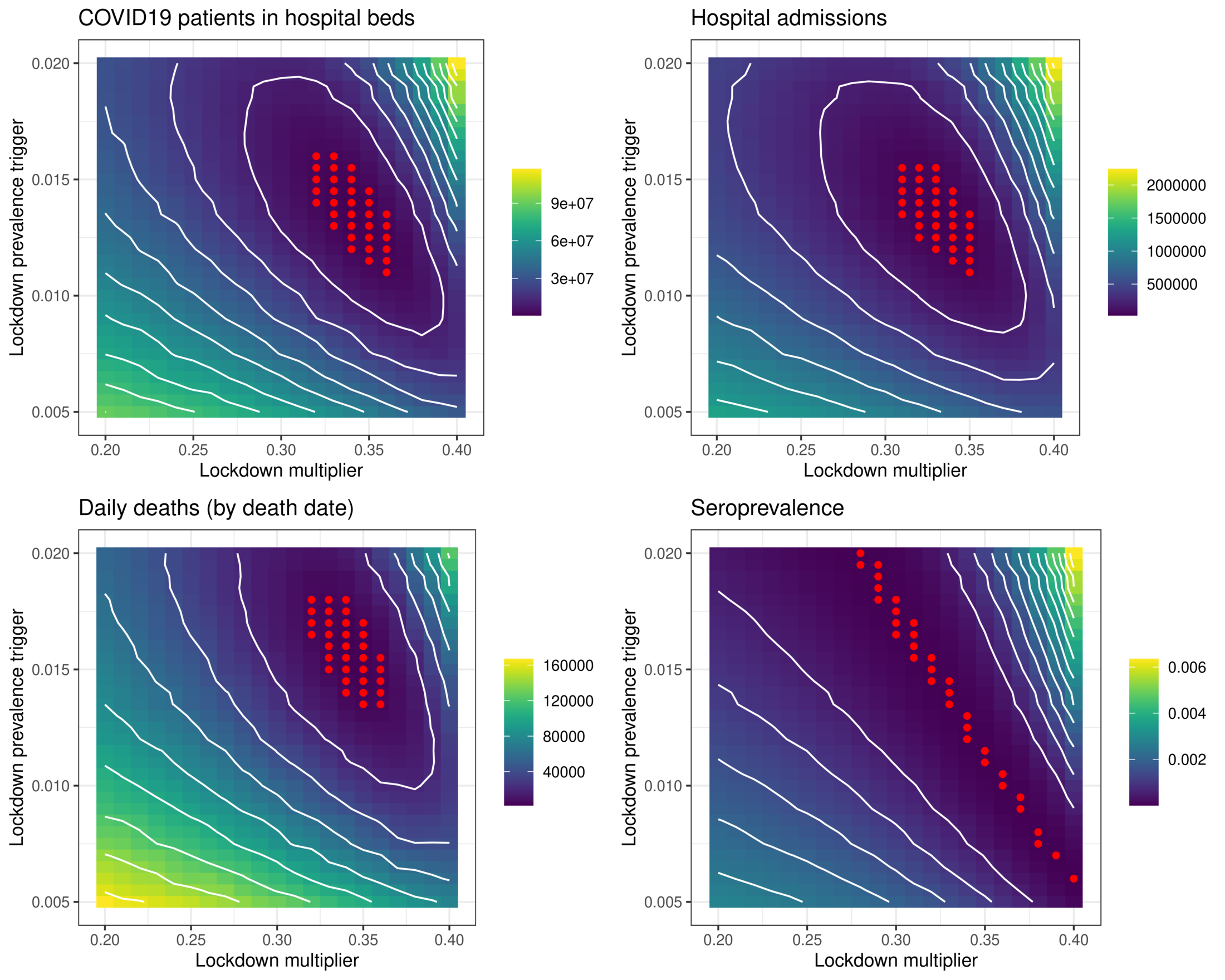

Supplement: S21 Fig — Simulations are of 56 million individuals, performed separately for a grid of values across a two-dimensional grid of 1) prevalence of SARS-CoV-2 at which lockdown was implemented (y axis), and 2) reduction in daily contacts during lockdown (x-axis). Surface has been interpolated from a grid of values. Transmission parameters (infectious_rate) fixed to assume a doubling time of approximately 3.5 days. Red dots highlight those parameter sets with the smallest 5% error with observed data. Observed data are from the UK Governments COVID19 dashboard and the UK’s Office of National Statistics (seroprevalence). (TIF) [file pcbi.1009146.s021.tif]

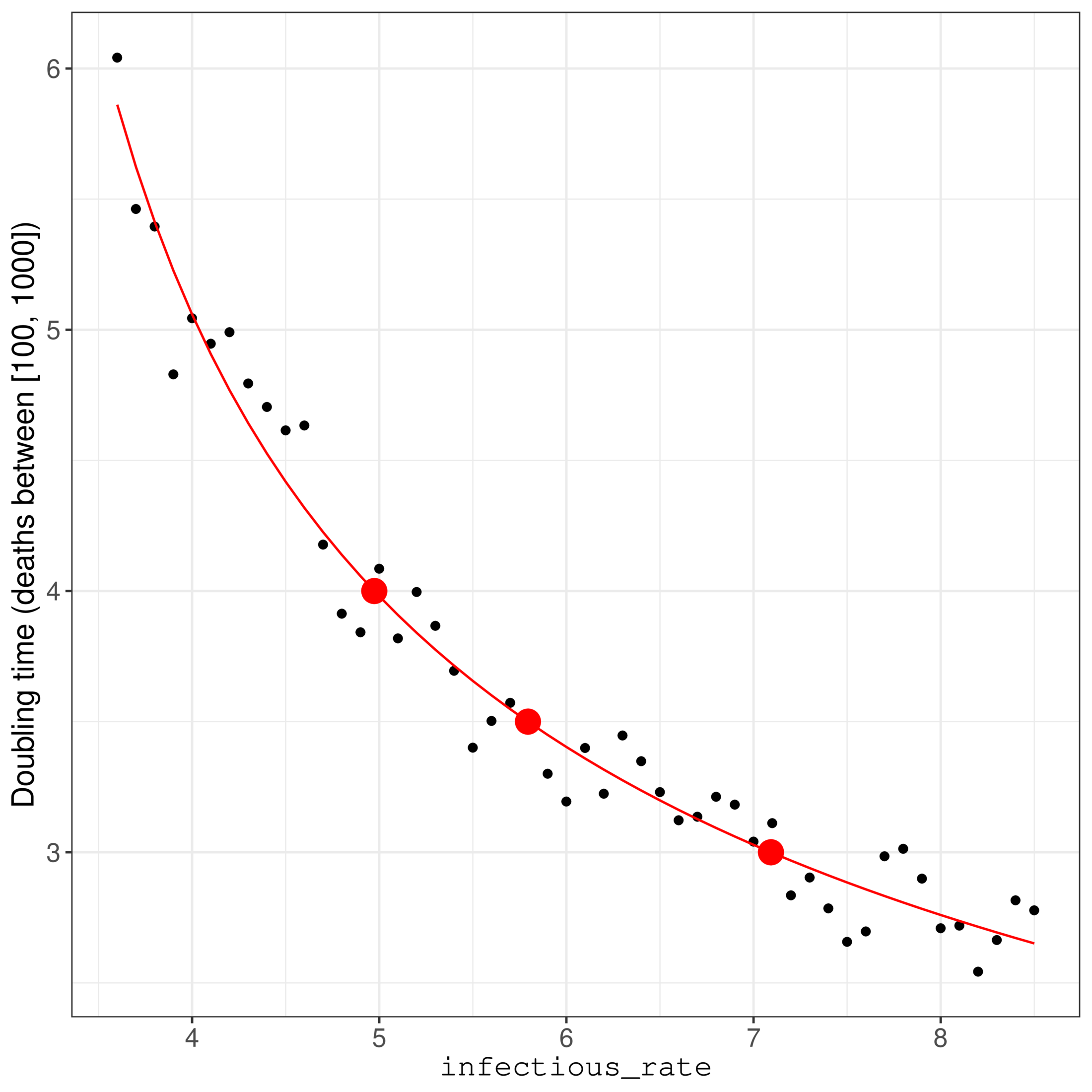

Supplement: S22 Fig — Simulations of 56 million individuals using OpenABM-Covid19 across a range of values of the infectious_rate parameter (black dots) across a range from 3.5 to 8.5 in increments of 0.1. Each black dot is the slope of fitting a linear regression to log of daily simulated deaths (truncated to between the first 100 to 1000 deaths). The red line represents a fit of the form a(x-b)^c * exp(nu), where nu is a noise term, to these data. Each red dot gives the value of the parameter infectious_rate (in brackets) for a doubling time of 3 (7.1), 3.5 (5.8), and 4 (5.0) days respectively. (TIF) [file pcbi.1009146.s022.tif]
