# Supplementary material for: OpenABM-Covid19—An agent-based model for non-pharmaceutical interventions against COVID-19 including contact tracing
Source: PLoS Comput Biol. 2021 Jul 12;17(7):e1009146. doi: 10.1371/journal.pcbi.1009146 (PMC8328312; doi:10.1371/journal.pcbi.1009146)
Supplement: Attachment — Submitted filename: OpenABM PLOS CB Response to Reviewers.pdf [file pcbi.1009146.s035.pdf]

24th April 2021

Dear Editors of *PLOS Computational Biology*,

We thank the Editors for the opportunity to resubmit our manuscript entitled "OpenABM-Covid19 - an agent-based model for non-pharmaceutical interventions against COVID-19 including contact tracing" to *PLOS Computational Biology*.

We would also like to thank the three reviewers of the manuscript for their helpful comments and suggestions. We believe these suggestions have resulted in an improved manuscript and description of the OpenABM-Covid19 model. We have addressed these comments and provide detailed responses below.

Thank you again for your consideration and we look forward to hearing from you in due course.

Best regards,

Dr Rob Hinch and Dr Will Probert, on behalf of all co-authors.

Below is the detailed response to the reviewers comments.

Line numbers refer to the marked up manuscript.

Reviewer #1: *The manuscrit describe a new computing agent-based code to simulate pandemic evolution, and to study some non-pharmaceutical interventions impact on this pandemic evolution. This approach allow to include some complex phenomena, such as heterogeneous social interactions, impact of physical distancing on the contaminations, contact tracing, social interaction in schools, at home, public transport, etc. This open-source code is writtend in C with a Python interface to allow various interactions between the user and the code, and for results display.*

The authors are invited to add in the manuscrit some clarifications of the limitation of their model (~ line 391), and some precisions, as described bellow.

- the authors use full randomized contact network to simulate public transports (line 157). What is the impact of such approach on "real" contacts, where people have some habits ? Is a partially-randomized network feasible

Response[1]: In reality, contacts vary in their regularity on a spectrum, from those that occur every day to those that are just a one-off. By default, OpenABM has three different networks to capture three points on this continuous spectrum: daily contacts within households, partially re-occuring contacts within our 'workplace' network, and contacts that almost never re-occur within the 'random' network (drawn randomly from the whole population each day). OpenABM has the ability to run on any network which can be imported via the Python or R interface, so users can explore using their own networks. An example of this is now added to the Supplementary Materials.

Manuscript Changes: Lines 590, 1100

- are the different interactions temporaly splitted to avoid overlap ? If no did you study the impact of the order of the different daily interaction computing on the evolution of the pandemy ?

Response [2]: All interactions are assumed to occur at the same time on each day. Given that, at the point of becoming infected, people are not instantly infectious, then the precise order of interactions on a day is not important.

Manuscript Changes: None

- line 206 : 65 millions people simuled by aggregating 65 simulation of 1 million people. Are 65 non-interactive simulations similar than a simulation of 65 M agent where everybody can interact with everybody ?

Response [3]: We've added a new figure in the Supplementary Materials to address this question. A study was done on looking at the variation of key statistics (doubling time/total infected) for different sized populations and subdividing them into different numbers of subpopulations. The analysis showed that the mean value of these key statistics was approximately independent of population size. Further, with a population of 1 million agents, the stochastic variations in these key statistics was minimal. Additionally there was no difference in the size of stochastic variation between running a simulation on 1 million agents or aggregating across subpopulations which had a total population of 1 million agents.

Manuscript Changes: Lines 251, 1110

- is an homogeneous spatialized distribution of people representative of real demographic distribution, where some regions are more dense than other ? (in particular for public transport and number of daily social interactions)

Response [4]: Answer similar to [3].The networks used are representative of the different types of interactions but are not intended to contain detailed geographical networks. Locally clustered interactions are included in both occupational and household networks.

Manuscript Changes: None

- what are the data used for model calibration ? Number of death ?

Response [5]: There are four pieces of observed data from the first wave of the COVID19 epidemic in England to which model predictions are compared over a grid search of parameters. Three pieces of data are from the UK Government's Covid19 dashboard (https://coronavirus.data.gov.uk/): deaths (by date of death), hospital admissions, and COVID19 patients in hospital beds. Seroprevalence estimates for England are taken from the UK Office of National Statistics (6.5% on 8th June 2020).

This figure (fig 5) is an illustration of the utility of the priors on the parameters (highlighting that very minimal calibration is needed in order to recreate the dynamics of the epidemic in England in several indicators). It is not meant as a detailed calibration approach.

Manuscript Changes: The description of the calibration has been updated in the main text and two figures highlighting the choice of parameter values have been added to the Supplementary Material (figs S22, S23).

- line 270 : the lockdown is taken into account with a reduction of socials interactions of 71%. Is this value deduced to fit to the mesures or taken from a previous study ?

Response [6]: These values are fitted using a parameter grid search (they are multipliers that are applied to the number of daily contacts on several networks). However, this estimate is in line with survey data from the first wave of the epidemic in England that suggests social contacts were reduced by 74% (Jarvis et al., 2020).

Manuscript Changes: We have described the calibration to the epidemic in England in more detail and cited the study of Jarvis et al., 2020.

- line 283 : did you deduce the probability of transmission with and without physical distancing ?

Response [7]: The overarching transmissibility (the "infectious_rate" parameter) is calibrated in the default parameter set by acknowledging the doubling time of the UK epidemic was approximately 3.5 days. The "lockdown multiplier" parameters in the model determine reductions in daily contacts (physical distancing) when interventions such as lockdowns are implemented. We have calibrated these parameters to fit to seroprevalence, deaths, hospital admissions in the first wave of the epidemic in England (see updates to figure 5 and S22 S23). Users can specify these parameters as they wish.

Manuscript Changes: A more thorough description of the calibration has been included.

- line 431 : every individual interact with each other every day. Same queestion than before : is this temporally splitted to avoid overlap contamination (A <-> B <-> C contribute each day of the contamination A <-> C) ?

Response[8]: People are not infectious on the day they become infectious, therefore it is not possible for an infection to go from A->B->C on a single day, so the ordering on a day is not relevant.

Manuscript Change: None

- line 504 : is the 2 factor used is deduced to fit to the observed data or is this documented ?

Response [9]: The parameter was chosen to increase the secondary household attack rate which was too low without this adjustment. The model's secondary household attack rate is 25%, which was calculated by summing the total number of household infections from the first person infected in a household divided by the sum of other people in those households. This compares well with estimates from Germany (21%), the US (24%) and the Netherlands (28%); although estimates from China have been lower. Given the UK focus of the default parameterisation, we used the European/US estimates.

Manuscript Changes: Lines 241, 625, 893

- line 600 : is the code parallelized (OpenMP or MPI) ? Did he scales correctly (in term of computing time / number of agents, and in term of number of core used if paralelized) ?

Response[10] - The basic model is not parallelised, however, we do have a meta-model which runs the underlying model on sub-populations and then allows cases to be seeded between populations at the end of each time-step. This is implemented in Python using the multiprocessing module.

The Performance section has been completely rewritten to reflect this and includes a figure on scaling.

Manuscript Change: Line 695, 722, 738, 892, 1105

- line 600 : 1.7 Gb memory for 1 million agents means ~ 400 simple precision (or ~ 200 double-precision) values used per agent without contact tracing. What are the main memory consuption to need so much RAM ?

Response[11] - since the initial submission, work on the code has reduced the memory requirement to 1kb per person. This is split approximately in thirds between storing interactions, networks, and data about individuals. Due to the variable and dynamic number of interactions each user has, interactions are stored as linked-lists requiring 20 bytes per interaction per person (2 longs for pointer to the other individual and the next interaction and 2 shorts for data about traceability of the interaction). Networks need to be stored in addition to interactions because we down-sample them to generate each day's interactions, with each edge requiring 2 longs (16 bytes).

The Performance section has been completely rewritten to reflect this and includes a figure on scaling.

Manuscript Change: Line 738, 1105

- due to high stochastic characteristics of a pandemy evolution (due to the random contacts between agents, and the random contagion from an agent to another), did you study the reproductibility of the simulations, and the range of results obtained with exactly the same parameters ? Some others agent-based approach, which use fixed contact-network, can show a wide variation of the max number of infected people, only due to the random aspect of contagion at each social contact. A study on these variations needs to be added to the manuscrit (These effects are visible for initial conditions with low number of infected people). The results used for decisional help are they an average of many simulations, or only one-shot simulation, without any error bars due to the stochastics effects ? This clarification is imperative, and may need to balance the conclusions obtained by the simulation described in the manuscrit.

Response [12]: See Response [3] and the accompanying figure which is a study covering the questions asked here.

Manuscript Changes: Lines 251, 1110

- what are the initial conditions ? Some infected at t = 0 ? How many ?

Response [13]: A small number of individuals are infected at t=0, parameter is n_seed_infection which is set to 5 (see S6 Table).

Reviewer #2: *The paper describes the implementation of an ABM for simulating the spread of Covid-19 within a population of up to 1 million people, although as it is described this number could be increased. Spreading occurs through both static and dynamic contact networks using statistics on contact patterns, which addresses the inhomogeneous spread in different age-groups. Also, disease progression and asymptomatic cases are modelled. The strength of the presented simulation model is the inclusion of a range of different prevention measures in addition to the classic NPIs for contact reduction. As this is a core advantage of this implementation, systematic assessment and testing of intervention strategies should be performed and discussed more deeply.*

• The authors state that the model is parameterized for the UK, however, the paper lacks information on the calibration and validation process and how well the model reproduces the historic epidemic curve and efficiency of actual NPIs.

Response [14]: We have now included a more detailed description of the calibration process in order to obtain the basic fit for the first wave of the epidemic in England.

Manuscript Changes: A more thorough description of the calibration has been included in the Methods. Figures S22 and S23 have been included to justify these parameter choices. We have also cited the study by Jarvis et al., (2020) highlighting the efficacy of the simulated lockdown after calibration is in line with other studies.

• The model seems to be able to reproduce the characteristics of a certain (?) time interval of the COVID epidemic in the UK. Line 229 "The model provides a good fit to UK data, correctly matching: the cumulative number of deaths; the magnitude of the peak in daily deaths; the timing of the peak in daily deaths to within a few days; and peak hospitalisation to within 25% of the recorded number" This could be supported with additional quantification of errors/correspondence in the comparison between real data and simulation results. How was this correspondence achieved? How good is the quality of fit compared with other models? Several times in the text the authors mention that the model (and parameters) were calibrated, but a description or discussion of the calibration approaches is not found in the manuscript.

Response [15]: As per response [14], we have now included a more detailed description of the calibration. Figures S22 and S23 justify parameter choices and error calculations against observed data.

Manuscript Changes: More detailed description of the calibration and additional figures (S22 and S23).

• The line "The model was run on a population of 65 million people with UK demographics, by aggregating 65 simulations of 1 million people. An infection was seeded and grew exponentially with a doubling time of 3.5 days." It should be described why does the model fits nevertheless? Based on the methods, the doubling time is a result of the simulation. This should be explained and why is it (?) constant?

Response [16]: The doubling time of 3.5 days has been used as a part of the calibration to determine a suitable value for the infectious_rate parameter from the early exponential growth. We have added a more thorough description of the calibration approach, including the doubling time, and two figures to highlight different choices of this infectious_rate parameter for different doubling times.

Manuscript Changes: Details on the calibration and two figures on the calibration in the Supplementary Materials.

• Paragraphs with important "messages" should be revised thoroughly. To give a more or less random example in line 600 ff "Performance. The ABM for 1 million individuals takes approximately 3s per day to run…" It is described in the abstract, but missing at this point for

what scenario and which time range. Such shortcomings can be resolved easily and can improve the quality of the paper to a large extent.

Response [17]: The section on Performance has been completely rewritten and an additional figure has been added. See Response [11] for details.

Manuscript Change: Line 738

• Evaluation of results of prevention measures is provided in supporting information only (see comments below). The analysis and assessment of interventions should be systematic with visual displays supporting a thorough discussion. In the current state, intervention scenarios are somehow treated as technical demonstrations.

Response[18]: The characterisation that the interventions are presented as technical demonstrations is intentional. The aim of this paper is to present a detailed description of both the model and the software interfaces. A systematic analysis of digital contract-tracing strategies has now been published in an article in Nature Digital Medicine. We have added a new paragraph at the start of the Results section outlining both this study and the use of the model by the NHS in the UK. (also see Response [39]).

Manuscript Changes: Lines 160, 905

• The abstract description of the model is well written and insightful, but technical details and modelling decisions are not or provided. The appendix is very useful and the parameters are well described. Even though established approaches in an agent-based simulation of epidemic spread and the state of the art (and its limitations as a motivation for the paper) should be recognized (if it is the goal of the paper to show the methodological improvement). The motivation for modelling decisions and assumptions should be discussed in the presentation of a simulation model. E.g. motivate the use of certain types of networks/graphs and probability distributions! What are the features of the specific mathematical concepts? Why are they suitable to model certain aspects?

Response [20]: Our model was developed first to support the development of digital contact tracing, to support the development of the NHS COVID-19 app, and then has evolved in response to stakeholder requests, from the NHS, from different government departments seeking advice on policies around testing and tracing, and from international stakeholders. A particular strength of our model is the ability to include different types of contact tracing, and it's coupling to realistic epidemiology. This paper presents the model structure which many users have already found convenient for different types of applications, and we feel that a detailed description of the epidemiological questions is out of scope in what is already a long paper. Examples of OpenABM-Covid19 being used to analyse epidemiological questions are now cited in the Results section. We have addressed many of the more technical points throughout our revision.

Manuscript Changes: Lines 160, 905

• The paper lacks technical details on implementation for assessing whether the efficiency claims hold, however as it is open source it is possible to review the code itself.

Response[21]: The Performance section has been completely rewritten to reflect this and includes a figure on scaling.

Manuscript Change: Line 738

• A technical overview of the implementation as a simulator/framework should be provided. E.g. what is an "object-oriented programming style"? To the knowledge of the reviewer, C is not an object-oriented programming language. What is the procedure for sampling a population? A description of the initialization phase would be helpful. How are individuals aggregated into households on a technical level? How are the networks sampled from data? This should be included despite the source code is publicly available and well documented.

Response[22]: The comment on OOP is addressed in Response[41]]. A couple of additional sentences have been added to the *Methods/Interaction Network* section to give more details on how the households/individuals are initially sampled.

Manuscript Change: Line 548, 701

• In the discussion, a kind of outlook is included, which should be described in more detail. A focus is set on hospitals, but big importance on epidemiological modeling will be set on the possibility to include also pharmaceutical interventions like vaccination

Response [23]: Since the initial submission, vaccinations have been added to the model. This is now included, see Response[38] for details.

Manuscript Changes: Line 39, 484, 1126, 1137

• How is the model dealing with "unreported cases"? As it is immanently clear, this aspect should be at least mentioned.

Response [24]: The time-series output of the model reports both total infections (regardless of test status) and total cases (only those who have tested positive.

Manuscript Changes: Line 392

The simulation model is of high quality and shows also high potential. But at the current state, the paper is a conceptual presentation of a simulation model but does not include or present interesting results on one of the three areas 1) epidemiology, 2) HCI and Usability, or 3) technical novelty. The paper should increase focus towards one of those directions. If the

model can be adapted as easily as claimed by the authors the model could provide a good framework for additional research for the assessment of NPIs if the model is correctly parameterized for the addressed research question.

Response [25]: The aim of the paper is to describe the model and its interfaces, and to demonstrate how it can be used to analyse interventions (i.e. your point 2). We believe the strength of our model is its usability, robust code and ability to be extended. To strengthen the work in this direction we have added an R interface to make it usable to a wider audience. A detailed analysis of the performance of the code has been added. Finally, vaccinations have been added to the model showing how easily it can be extended (this feature is being used by NHSE to analyse the effect of the UK vaccine programme on hospital admissions). Whilst this paper does not contain a detailed assessment of a precise epidemiological research question (i.e. your point 1), we now describe and cite work where OpenABM-Covid19 has been used to do this.

Manuscript Changes: Lines 44, 65, 454, 486, 738

Some of the figures in the manuscript look not very appealing. For instance, Fig1 could be supported by context if placed in the Methods section. The inclusion of parameters is important. All mathematical symbols should also be used in the parameter tables. It would be good to provide additional context to the parameter values with some of the supporting tables.

Response [26]: Thank you for the comment. Several of the figures in both the main text and supplement have been updated which we hope are now more appealing. We agree that parameters need good documentation and so the symbols for all parameters mentioned in the documentation are provided in the parameter dictionary in the model documentation on the github repository: https://github.com/BDI-pathogens/OpenABM-Covid19/blob/master/documentation/parameters/parameter_dictionary.md

Manuscript Changes: Updated figures.

The model is fully documented and is thoroughly tested. The formal testing framework (mentioned in line 358) could be described in more detail e.g. line 589 "The model codebase includes over 200 tests used to validate the model." What was included in the tests? How was validation implemented?

Response [27]: Thank you for this comment. The pytest framework, as mentioned in the main text, takes model outputs and compares them against expected values for known input parameters. Every new feature or piece of code offering new functionality has to include tests and the tests need to pass in order to be merged into the main repository (https://github.com/BDI-pathogens/OpenABM-Covid19/blob/master/CONTRIBUTING.md). We have highlighted this in the main text. Tests cover all major areas of the model - disease dynamics, infection dynamics, setting parameter values, and control interventions. As

mentioned in the text, there are over 200 tests and documenting them all in the main text would not be possible.

Manuscript Changes: None

Model descriptions and modelling process could be set concerning international standards or guidelines like "Modelling Good Research Practices of ISPOR-SMDM" (https://www.sciencedirect.com/science/article/pii/S109830151201652X) . For example "V-8 If using an agent-based model, thoroughly describe the rules governing the agents, the input parameter values, initial conditions, and all sub-models."

Response [28]: We agree that agent-based models need to be rules for agents should be thoroughly described and parameter values/initial conditions documented. OpenABM-Covid19 is thoroughly documented both on Github (where it has been developed as an open source project) and now described in this paper. All parameters are documented on Github and have been reproduced as Supplementary Tables in this paper.

Reviewer #3: *This paper presents a detailed description of the OpenABM-Covid19 model, which is an agent-based COVID-19 transmission model informed by detailed data on contact networks and validated against data from the UK. As a methods paper, it does not include results per se, but rather illustrates the analyses the model can be used for.*

Overall, I found the paper to be exceptionally well written and clearly laid out. The model has been carefully conceived, and the use of modern software practices (testing, documentation, concern for adaptability, an easy-to-use Python user interface, etc.) make this study an exemplar for how such models should be developed and communicated. The following comments are mostly intended as nonbinding suggestions for improving the paper.

p. 4, line 56: This could be interpreted to mean that the population size fixed at 1 million, rather than that 1 million is the default.

Response[29]: Agreed, reworded to make clear this a default population size.

Manuscript Changes: Line 57

p. 5, line 85: I'm not sure I understand how people who are contact-traced are themselves "protected" -- wouldn't they be contact traced following exposure to a known positive?

Response [30]: Due to the clustered nature of social networks, infections tend to be clustered on the network. Therefore, if you have been contact-traced by one of your contacts who is infected, then it is likely that you have other contacts who are infected. These people may be asymptomatic or not isolating, therefore if you contact-traced and quarantine yourself, you are protected from these other locally infected people on your social network.

Manuscript Changes: None

p. 6, line 96: Quite a few groups have developed COVID ABMs; while a comprehensive literature review is probably beyond the scope of the introduction, additional citations of influential ABMs might help the reader better understand the modeling landscape. The following are suggestions only:

** The Imperial model, which was influential in UK policy decisions (https://www.imperial.ac.uk/media/imperial-college/medicine/sph/ide/gida-fellowships/Imperial-College-COVID19-NPI-modelling-16-03-2020.pdf)*

** Blakeley et al., which was influential in Australian policy decisions (https://www.mja.com.au/journal/2020/213/8/probability-6-week-lockdown-victoria-commencing-9-july-2020-achieving)*

** Koo et al. (https://www.thelancet.com/journals/laninf/article/PIIS1473-3099(20)30162-6/fulltext)*

** Aleta et al. (https://www.nature.com/articles/s41562-020-0931-9)*

** Rockett et al. (https://www.nature.com/articles/s41591-020-1000-7)*

In addition, the specific claims about OpenABM-Covid19 in comparison to other models may not be entirely accurate. Rockett et al. and Bicher et al. used 23 million and 9 million agents, respectively, which are larger populations than are typically used in OpenABM-Covid19. In addition, my understanding is that Covasim's computational efficiency is comparable to OpenABM-Covid19 (1 second per 2 million person-days; see Fig. S6 of https://www.medrxiv.org/content/10.1101/2020.07.15.20154765v4.full.pdf), and has also been designed with extensibility and ease of development in mind (e.g. 100 forks on GitHub). The authors are encouraged to rephrase in a way that emphasizes the strengths of OpenABM-Covid19 while also acknowledging the strengths (and weaknesses!) of other models. (Disclosure: I am one of the authors of Covasim.)

Response [31]: Thank you for this suggestion. We have included the mentioned citations, which we agree will help the reader orient themselves in the COVID19 ABM landscape.

Manuscript Changes: p6

p. 7, line 111: While I can see a (strong) argument for community and perhaps workplace contacts to be drawn from a negative binomial distribution, is this true of household and school contacts as well? We have found that overdispersion in infectiousness, rather than overdispersion in number of contacts, is the most important factor for driving superspreading events. If in your model the latter are alone sufficient to account for the observed distribution of secondary infections, this is an interesting finding!

Response[32]: Only the random (community) contacts are drawn from the negative binomial distribution. Household contacts are constructed using census data and occupation contacts are constructed using small-world networks (where the number of contacts is age dependent). Details are in Methods/Interaction Networks.

We have quantified the super-spreading events predicted by the model and have noticed that the network alone does not provide sufficient overdispersion to explain this (k=2-3). So, we've added an individual infectiousness multiplier to the model which is drawn from a gamma distribution with mean 1. We've added a figure which shows the offspring distribution with a MLE fit of a binomial to estimate k(=0.5) which is now in range of the published estimates from empirical studies. Thank you for pointing this out.

Manuscript Changes: Line 231, 626, 638, 920, 982, 1142, 1148

p. 8, line 139: Out of curiosity, is there any reason why this version cannot be considered 1.0? The codebase seems mature, tested, and documented, and the bulk of development seems to have been completed >6 months ago, which would seem to exceed the threshold for a 1.0 release in most contexts. (Very minor point: the Python package installs as version 0.2, not 0.3.) While acknowledging that the software practices are light-years ahead of most models, I did find myself wishing for a changelog, at least for backwards-incompatible changes (i.e. if the same parameters would no longer run, or would give a different result).

Response [33]: Thank you for this suggestion. We have now called this version, in line with the publication, version 1.0.

Manuscript Changes: None. Model repository has been adjusted to use version 1.0.

p. 10, Figure 2:

1. For Fig. 2A, showing the three distributions separately might be easier to read (as is, it looks like some of them are negative).

2. I am also surprised at how low the number "random" contacts are -- I assume this does not count the 50+ people one would be in "contact" with on the metro or at a grocery store.

3. I didn't realize until getting to the Methods section that school students were included in the "occupation" network. This surprised me since school class sizes and workplace sizes tend to have fairly different distributions (the former having a larger mean and smaller variance than the latter). In addition, given the central importance of school closures as a COVID policy measure, including school networks explicitly would seem to be desirable. Unless this can be added quickly, it could be noted as a limitation of the model.

Response [34]:

1.We have amended figure 2A as suggested.

2.The number of contacts modelled is those with whom you have reasonable contact, so within 2m for at least 15 minutes. Therefore, it represents the people you sat next to on the bus/train as opposed to the entire bus/carriage.

3.The occupation networks are separated by age, so schools are represented by the “occupation” networks with the lowest age. These do have a higher number of mean interactions than the adult and elderly occupation networks to model the higher number of interactions.

p. 11, line 208: Typo, "day"

Response [35a]: Typo fixed.

Manuscript Changes: Line 274

p. 11, Fig. 4: Perhaps a few words could be said about how the model-derived IFR compares to empirical estimates, e.g. Ferguson et al. (which seems like it was used for some of the input parameters), O'Driscoll et al. (https://www.nature.com/articles/s41586-020-2918-0), and/or Brazeau et al. (https://www.imperial.ac.uk/mrc-global-infectious-disease-analysis/covid-19/report-34-ifr). By eye at least, the match looks quite good (i.e., at least as consistent as these estimates are with each other), which is nice evidence for model validation.

Response [35]: Thank you for this suggestion. We have now highlighted the concordance with the model-derived IFR and the published studies on the age-stratified IFR.

Manuscript Changes: p14

p. 13, Fig. 5:

1. What process, if any, was used for calibrating the parameters to produce these outputs? It seems some relatively straightforward tuning would be able to produce a better fit (e.g., lowering the infection hospitalization rate; increasing the hospitalization mortality rate). But if no tuning was done at all to parameters, the fact that the parameter "priors" produce such a good fit is worth highlighting.

2. X-axis labels are missing -- I assume this is days? If actual date labels could be used, it would make it easier to read.

3. It would be interesting to know how well the model can fit the UK's 2nd and 3rd waves, but one can easily get lost in an infinite spiral of fitting the model to the latest data, so I would not consider doing this a requirement.

4. Does the model produce estimates of diagnoses, and if so, would it be possible to see the projections for these as well?

5. Some indication of uncertainty would be valuable -- stochastic uncertainty if not parametric uncertainty. I understand that this will be highly dependent on the number of seed infections used, however.

6. The commit hash mentioned here does not match the previous one, even though this figure seems to be the central result of the paper. Does the previously mentioned commit hash refer to the IFR results? Would one get different results if running with a different commit hash (e.g., 536adae at the time of writing)?

Response [36] and Manuscript Changes:

1. There is very minimal calibration to produce these figures. Three parameters were varied: 1) the infectious_rate parameter, 2) the prevalence in the population at which lockdown was implemented, and 3) the reduction in the number of daily contacts during lockdown. The infectious_rate parameter was calibrated to fit a doubling time of 3.5 days of the first 100 to 1000 deaths. The other two parameters were fitted to deaths, hospitalisations, patients in hospital beds, and seroprevalence. The purpose of this figure is not to illustrate a particular calibration technique but, as highlighted by the comment, illustrate that the parameter priors provide a reasonable starting fit to the data. Thank you for raising this.

2. The X axis is days after lockdown (all simulations are aligned on 23rd March 2020 when lockdown began in the UK). We have now added dates to the X axis.

3. Thank you for raising this. We are hoping to showcase a flexible model rather than a calibration framework. The model can be paired with a range of different calibration frameworks and we invite other researchers to pair the model with whichever calibration framework they are most comfortable with.

4. The model includes ‘cases’, as those that have returned a positive COVID-19 PCR test.

5. Thank you for raising this. We have now included stochastic uncertainty in the figures (i.e. fixed parameter values and run for several random seeds). Figure 5 includes 50 stochastic replicates.

6. Thank you for pointing this out. We have now removed commit hashes throughout the paper and only included a single version of the model.

p. 18, line 365: If I'm not mistaken, it has recently become possible to run from R as well as Python? (Although this seems less well documented.) This might be worth mentioning given the large number of epidemiologists who use R.

Response [37]: Thank you for suggesting this. There is now an R interface available for OpenABM-Covid19. It is not currently on CRAN (but will be soon) but there are instructions for installing the R version on the README.md of the github repository for the model.

Manuscript Changes: Line 44, 65, 454, 486, model code repository

p. 20, line 394: It is also probably worth mentioning pharmaceutical interventions, since I see vaccination has also been recently implemented in the model.

Respons [38]: Yes, this was added since the initial submission and is currently being used by the NHS in the UK to model the effect of the UK vaccine programme on hospital admissions. There are 2 vaccine models in OpenABM, one which protects fully against infection and one which prevents symptoms developing. This has been added to the list of interventions, along with a Supplementary Figure demonstrating the effects of the 2 types of vaccines and the R code required to generate it.

Manuscript Changes: Line 437, 1126, 1137

p. 20, line 403: I was surprised there was no mention in this paper of the contexts and applications OpenABM-Covid19 has been used for. I feel silly saying this, but citing https://www.medrxiv.org/content/10.1101/2020.08.29.20184135v1 would seem to be needed at minimum!

Response [39]: We have added an extra paragraph at the start of the Results section discussing the uses of OpenABM-Covid19 in the context of public health in the UK and the study in Washington State (now published in Nature Digital Medicine).

Manuscript Changes: Lines 160, 905

p. 23, line 468: It sounds like some individuals are assigned a high daily number of contacts and persist with that number of contacts. It may be more realistic to redraw the number of contacts per person each day as well since superspreading events tend to happen at non-daily venues (e.g. churches, restaurants). However, this is unlikely to make much difference to the results. In addition, it would be interesting to see the distribution of primary vs. secondary cases, such how well the model matches the data that e.g. ~20% of people are responsible for ~80% of transmissions.

Response [40]: Whilst we agree that there will be variation in the number of contacts a person has each day, our expectation is that people who have a large number of random contacts one day tend to have a large number of random contacts every day due to persistent commuting/behavioural patterns. Therefore, we expect that keeping the number static from day to day is a better model than allowing agents to jump from low to high numbers. Of course, there are non-daily one-off events such as those mentioned by the reviewer, however, that is a level of greater realism in the social networks than we are modelling. The amount of super-spreading is now quantified and the model has a k-value of 0.5 which is inline with the literature (see Response [32]).

Manuscript Changes: Line 231, 626, 638, 920, 982, 1142, 1148

p. 28, line 571: I am curious to know why it's claimed that the approach is object-oriented -- C is not generally considered an object-oriented programming language (https://softwareengineering.stackexchange.com/questions/113533/why-is-c-not-considered-an-object-oriented-language). I see structs being used extensively to handle data of different types, but I don't see much use of pointers to functions being used to emulate class methods, for example, and as such it looks a bit more functional to me. (Of course, one could "just" recode the whole thing in C++!)

Response [41]: We have changed the language to say that only the Python and R interfaces are OOP. We use the wording "object-oriented coding style" as opposed to OOP since, as noted by the referee, C lacks the formal object structure. However, the functions were written as if they were class-methods with the first argument being the structure (class) which they operate on. This meant when the C-code was interfaced with objects in Python/R, there is a one-to-one mapping between the class-members and the C-structure; and class-methods and the C-functions.

Manuscript Changes: Line 701

p. 28, line 579: It might help to explain what SWIG is (I had to google it).

Response [42]: We have added a sentence about SWIG and a reference.

Manuscript Changes: Line 710, 891

p. 29, line 603: Is it possible to disable rebuilding the daily interaction network? I imagine this would increase performance by perhaps an order of magnitude, and can approximated as a larger network with lower transmission probabilities (i.e., 10 new contacts per day for 5 days each with a 1% transmission probability is virtually identical to 50 static contacts for 5 days with an 0.2% transmission probability -- given the wide uncertainty bounds of how many contacts should exist in the first place).

Response [43]: Yes, we have implemented the ability to have static-network where the contact patterns are kept static each day. As predicted, this dramatically increases the performance, with the per-time-step time being reduced to 50ms for a population of 1m people and 15ms for the meta-model with 1m people (on a quad-core machine). This is explained in detail in the new Performance section.

Manuscript Changes: Line 695, 722, 738

p. 52, line 814: Perhaps consider a protected branch instead of a commit hash, e.g. https://github.com/BDI-pathogens/OpenABM-Covid19-model-paper/blob/ploscb/notebooks/example_digital_contact_tracing.ipynb

Response [44]: Thank you for this suggestion. This repository housing the code for reproducing the analyses in this paper is now in sync with the paper figures and we have therefore avoided references to any commit hashes on this analysis repository.

Manuscript Changes: Changes to the repository housing the analysis.

References

Jarvis, C.I., Van Zandvoort, K., Gimma, A. *et al.* Quantifying the impact of physical distance measures on the transmission of COVID-19 in the UK. *BMC Med* **18,** 124 (2020). https://doi.org/10.1186/s12916-020-01597-8
